# A rationally designed 18-amino acid peptide with potential as GLP-1 receptor agonist

**DOI:** 10.3389/fphar.2026.1789257

**Published:** 2026-05-20

**Authors:** Aditi Singh, Sucharita Shadangi, Soumendra Rana

**Affiliations:** Chemical Biology Laboratory, School of Basic Sciences, Indian Institute of Technology Bhubaneswar, Bhubaneswar, Odisha, India

**Keywords:** DPP-4, GLP-1RA, insulin resistance, pancreatic β-cells, T2DM

## Abstract

**Introduction:**

Diabetes mellitus (DM) is a multifaceted disease etiologically characterised by dysregulation in glucose homeostasis. The World Health Organization (WHO) global report indicates that over 90% of DM cases are classified as Type 2 DM (T2DM), which is clinically characterized by chronic hyperglycemia. This systemic condition arises predominantly due to the interplay of two key components: (a) compromised insulin production by the pancreatic β-cells, and (b) the failure of insulin-sensitive tissues to react to insulin. Notably, it is well established that glucagon-like peptide-1 (GLP-1), an incretin hormone of the glucagon superfamily, contributes to glucose-dependent pancreatic β-cell insulin secretion. The insulinotropic impacts of secreted GLP-1 are facilitated by its interaction with GLP-1 receptor (GLP-1R), a class B G-protein-coupled receptor (GPCR). However, GLP-1 is proteolytically cleaved by dipeptidyl peptidase 4 (DPP-4), resulting in a plasma half-life of ∼2 minutes, which limits its therapeutic efficacy in patients with T2DM. Therefore, the exogenous administration of DPP-4-resistant GLP-1R agonists (GLP-1RAs) has proven to be a successful therapeutic strategy for managing T2DM. Notably, the currently marketed GLP-1RAs, such as Semaglutide, Liraglutide, and Lixisenatide, are long-chain GLP-1 mimetic peptides, ranging in length from 33 to 39 amino acids.

**Methods:**

In this context, the computational design and in silico evaluation of a DPP-4- resistant, potent designer helical peptide agonist (SR18; ≤18 aa) of the GLP-1R, comprised of both coded and non-coded amino acids, are described in the current study. The basic pharmacological activity of the designer peptide, SR18 was evaluated through circular dichroism, dynamic light scattering, proteolysis, cytotoxicity and hemolytic experiments.

**Results and discussions:**

SR18 preserves several amino acids necessary for effective interactions with the GLP-1R, similar to those found in GLP-1, Liraglutide, and Semaglutide. Interestingly, the binding of SR18 also mimics the binding of small-molecule agonists of GLP-1R. Preliminary experimental studies confirm that synthetically prepared SR18 maintains an ordered, α-helical conformation under various solvent conditions and possesses the basic pharmaceutical properties desired of a potent lead peptide. Furthermore, compared with GLP-1 and Semaglutide SR18 exhibits stable interactions with GLP-1R over 1 μs of molecular dynamics (MD) simulations, with appreciable binding affinity and energy, supporting its viability as a potential alternative to the current long-chain GLP-1R peptide agonists.

## 1 Introduction

Type 2 diabetes mellitus (T2DM) is a chronic hyperglycemia-induced metabolic disease (Singh et al., 2025a), which accounts for ∼90% of patients clinically diagnosed with diabetes. There has been a notable increase in the number of patients with T2DM due to the global rise in obesity, driven by sedentary lifestyles and regular consumption of energy-dense diets (Gupta et al., 2024; Hussain, 2025). Chronic hyperglycemia contributes to insulin resistance, a metabolic condition in which the body initially fails to respond to the natural endocrine hormone insulin, secreted by the pancreatic β-cells. Eventually, in response to excessive glucose stress, the β-cells fail to produce sufficient insulin, leading to β-cell dysfunction and subsequent onset of a severe form of T2DM (Accili et al., 2025). Therefore, several classes of drugs, such as alpha-glucosidase inhibitors (AGI), amylinomimetics, biguanides, bile acid sequestrants, dopaminergic antagonists, DPP4 inhibitors (DPP4i), insulin and insulin secretagogues (IS), sulfonylureas, meglitinides, sodium-glucose cotransporter-2 inhibitor (SGLT2i), and thiazolidinediones (TZDs), including the glucagon-like peptide-1 receptor agonists (GLP-1RAs) have been made available for the management of T2DM (Tahrani et al., 2016; Drucker, 2025). However, in the process of controlling hyperglycemic conditions, antidiabetic drugs like insulin, insulin secretagogues, sulfonylureas, and meglitinides trigger hypoglycemia. Additionally, severe side effects from a few antidiabetic drugs, like TZDs, which cause congestive heart failure (Lago et al., 2007; Shi et al., 2023), and SGLT2i, which cause genitourinary infections, are also documented (Pishdad et al., 2024). Notably, among all classes of antidiabetic drugs, GLP-1RAs, a form of incretin (INtestinal secretion of INsulin) hormone-based therapy, offer additional advantages, such as inducing weight loss and preventing hypoglycemia symptoms. On the other hand, GLP-1RAs can also cause gastrointestinal side effects and injection site reactions, while carrying a higher price tag than other therapeutic options like metformin. Nevertheless, the clinical benefits of GLP-1RAs outweigh these drawbacks.

**FIGURE 1:**
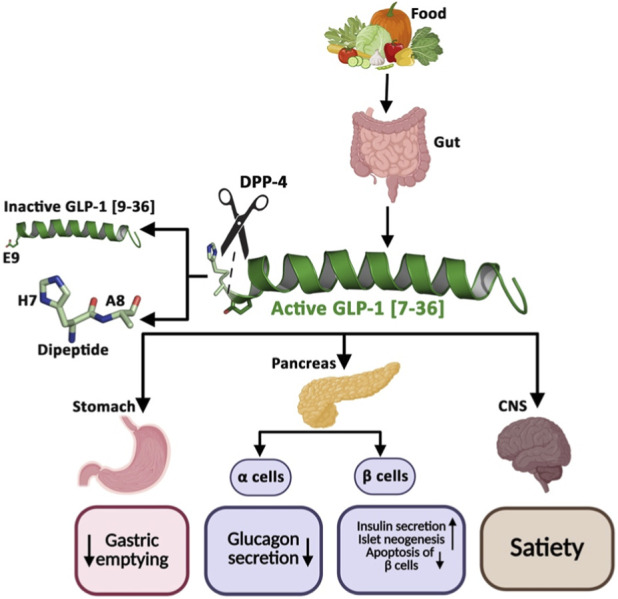
Schematic illustration of the role of GLP-1 in controlling postprandial hyperglycemia.

Glucagon-like Peptide-1 (GLP-1, 30–31 aa) is a natural gastrointestinal incretin hormone of the “glucagon peptide family”, secreted from the enteroendocrine L-cells in the distal gut to stimulate insulin secretion in response to postprandial hyperglycemia (Figure 1). Briefly, the preproglucagon gene undergoes tissue-specific post-translational modification with the help of specific propeptidase convertase (PC1/3) to produce two variants of GLP-1, the amidated GLP-1 [7–36], and the glycine-extended GLP-1 [7–37] in the ileum and hypothalamus (Lafferty et al., 2021; Zheng et al., 2024). However, the GLP-1 [7–36] amide accounts for 80% of circulating GLP-1 (Helmstadter et al., 2022), which lowers postprandial hyperglycemia through three established mechanisms: (a) deceleration of gastric emptying, (b) insulinotropic actions, and (c) suppression of glucagon function except for hypoglycemia (Hansen et al., 2024).

**FIGURE 2:**
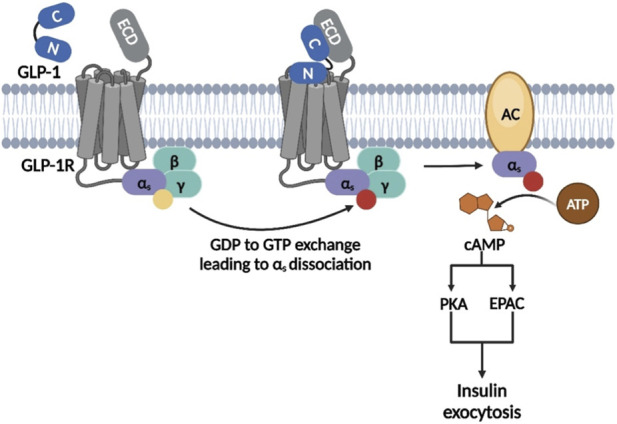
Cartoon representation of the “two-domain” binding interaction of GLP-1 with GLP-1R leading to insulin exocytosis.

Notably, the insulinotropic actions of circulating GLP-1 are facilitated through a “two-domain” binding interaction with GLP-1R (Figure 2), a structurally well-characterized prototypical class B GPCR, which recruits the Gα_s_-subunit of the heterotrimeric G-protein for cAMP-mediated downstream signaling, involving protein kinase A (PKA) and exchange protein activated by cAMP (EPAC), leading to insulin exocytosis. Briefly, while the extracellular domain (ECD) of GLP-1R, which is responsible for ligand recognition and specificity, engages the C-terminus of the GLP-1, the interhelical transmembrane crevice of the GLP-1R helps in docking of the N-terminus of the GLP-1, triggering downstream signaling (Fang et al., 2020).

However, the naturally secreted GLP-1 is quickly hydrolyzed and rendered inactive by dipeptidyl peptidase-4 (DPP-4), a transmembrane amino peptidase. This ubiquitous protease is expressed in several tissues, including pancreatic β-cells, and cleaves the amino-terminal dipeptide at the penultimate position with either L-proline or L-alanine (GLP-1), limiting the clinical utility of GLP-1 in T2DM patients, particularly its insulinotropic actions (Meier et al., 2006; Saini et al., 2023). The elimination half-life of the intact GLP-1, including the DPP-4-generated metabolites, such as GLP-1 [9–36] or GLP-1 [9–37]) is ∼2 min. Moreover, the DPP-4-generated metabolites of GLP-1 (Figure 1), lacking the His-Ala dipeptide, have ∼100-fold less affinity towards GLP-1R. DPP-4 also acts as a molecular bridge between obesity and T2DM. Several factors contribute to the expression and regulation of DPP-4 in the body. The extracellular domain of DPP-4, which harbors its catalytic site, often gets released from the membrane and circulates in plasma. The onset of obesity primes the enlarged visceral adipocytes and infiltrating macrophages to overexpress and release soluble DPP-4 in the bloodstream, where its levels correlate directly with BMI and insulin resistance in skeletal muscle cells and liver tissues. Elevated serum DPP-4 levels promote chronic inflammation, leading to the onset of T2DM (Ghorpade et al., 2018; Guo et al., 2024). Notably, some studies suggest that patients with T2DM have elevated circulating DPP-4 and lower plasma GLP-1 levels, both of which correlate positively with hyperglycemia and obesity (Lugari et al., 2004; Ryskjaer et al., 2006).

Nevertheless, the regenerative effects of GLP-1 on β-cells offer immense potential for T2DM patients, who may have lost nearly half of their β-cell activity by the time they were diagnosed (Shao et al., 2025). Therefore, GLP-1RAs that mimic the function of native GLP-1 but with improved pharmacokinetics are highly desirable as drug candidates for the management of T2DM (Prajapati et al., 2024). Thus, GLP-1 analogs are prepared considering two key points in mind: (i) Native GLP-1 is cleaved by DPP-4 at Ala8, and (ii) Arg or Lys residues are susceptible to serine protease cleavage, more specifically Lys34. Therefore, modification strategies, such as incorporation of noncoded amino acids (NCAAs), N-methylation, substitution with β-amino acids, backbone modification, and fatty acid acylation, have been employed to develop various GLP-1RAs to date (Wang et al., 2022). For instance, sequence remodeling and extension led to the discovery of exenatide (Byetta), a 39-amino acid synthetic peptide with ∼50% sequence identity to GLP-1, derived from exendin-4, a peptide found in the venom of the Gila monster *Heloderma suspectum* (Furman, 2012; Van Baelen et al., 2022)*.* The substitution of Ala8 in GLP-1 by Gly8 in exendin-4 contributes to enhanced DPP-4 resistance (Movahednasab et al., 2025). Notably, various exendin-4-based GLP-1RAs (Exenatide: 39 aa and Lixisenatide: 44 aa), and GLP-1-based GLP-1RAs (Dulaglutide: 31 aa, Liraglutide: 31 aa, Semaglutide: 31 aa, Tirzepatide: 39 aa, and Albiglutide: 60 aa) have been developed and approved by the FDA between 2005 and 2022 for the management of T2DM (Liu, 2024). In addition, other GLP-1RAs, such as Beinaglutide, Supaglutide, Emaglutide, and Efpeglenatide, are currently in clinical trials.

Interestingly, until now, primarily long-chain peptides with 33–39 amino acids have been developed as GLP-1RAs, which also carry a cost burden. Notably, non-peptide small-molecule agonists of the GLP-1R are also being developed for oral bioavailability (Knudsen et al., 2007; Kawai et al., 2020; Griffith et al., 2022). Therefore, there should be a reasonable effort to design alternative short-chain peptide-based GLP-1RAs with low toxicity and efficacy comparable to marketed long-chain peptides. In fact, designer peptides composed of nine to eleven amino acids have been developed earlier as potent GLP-1RAs that exhibit affinity and potency similar to the native GLP-1 (Mapelli et al., 2009; Jazayeri et al., 2017). Therefore, short peptides containing crucial amino acids required for interaction with GLP-1R can be designed to act as small-molecule GLP-1RAs for insulin exocytosis under hyperglycemic conditions. In this context, using a previously reported α-helical peptide as a template sequence (Behera et al., 2022), this study discusses the rational computational design and *in silico* evaluation of an α-helical peptide (SR18: 18 aa) as a potent agonist of GLP-1R, with native GLP-1 and Semaglutide serving as references. The analysis of molecular dynamics (MD) simulation data over 1 μs each for the GLP-1–GLP-1R and the SR18–GLP-1R systems, estimation of free energy of binding, including preliminary experimental studies on the synthetically prepared designer peptide, suggests that the designer peptide SR18 has strong signature traits of an agonist that can potentially trigger GLP-1R-mediated insulin exocytosis under experimental conditions in tissues.

## 2 Materials and methods

### 2.1 General computational methods

Protein Data Bank (PDB) coordinates of the GLP-1−GLP-1R (5VAI) (Zhang et al., 2017), Semaglutide (7KI0), Taspoglutide (7KI1) (Zhang et al., 2021), and liraglutide (4APD) were retrieved from the RCSB PDB server (www.rcsb.org). Multiple sequence alignments of the peptides were performed through Clustal Omega (Sievers and Higgins, 2018; Madeira et al., 2024). MODELLER (Webb and Sali, 2016), Discovery Studio (Biovia), and PyMOL (Schrӧdinger, Inc.) software were utilized for modeling, initial processing, visualization, analysis, and presentation of GLP-1R and model peptide structures. The data were plotted in GraphPad Prism (www.graphpad.com).

### 2.2 Molecular docking studies

The HDOCK web server (Yan et al., 2017b; Yan et al., 2020) was used to perform peptide-protein docking to predict the putative binding mode between the designer peptides and loop remodeled GLP-1R. HDOCK is based on a hybrid algorithm that combines template-based modeling and *ab initio* free docking. The PDB files of modeled GLP-1R and peptides were uploaded to the HDOCK server, and global docking was performed using the default parameters, which use a fast Fourier transform (FFT)-based rigid-body search method and then evaluate the interface energetics using an intrinsic scoring function (Huang and Zou, 2008; 2014; Yan et al., 2017a). For each docking run, the server generated the top 100 predicted complex structures, along with their HDOCK energy scores and confidence scores. Docking energy scores are calculated using iterative scoring functions ITScorePP or ITScorePR. This energy score indicates the expected binding affinity between the ligand and the receptor; more negative scores indicate higher binding affinity. The confidence score provides an empirical measure of binding likelihood between docked molecules. Confidence score >0.7 suggests strong binding, whereas a value <0.5 indicates poor binding (Li et al., 2022). The best conformation complex (with the highest HDOCK energy score) of the GLP-1R and the peptides was further screened for MD simulation. Additionally, the binding energies and the ∼K_d_ values of the top docking poses obtained from HDOCK were also estimated using PRODIGY (Xue et al., 2016).

### 2.3 Peptide design and synthesis

The sequence of the GLP-1 peptide and a few reported GLP-1RAs, like Exendin-4, Semaglutide, Liraglutide, and Taspoglutide, were first aligned to obtain the mutating residue. Further, one of the earlier reported model helical peptides (Hed3: NH_3_^+^-YGKAAAAK-Igl-AAAKAAAAK-CO_2_^−^) was utilized to generate the template GLP-1 mimetic peptide (Hed6: NH_3_^+^-H-Igl-E-Aib-AAAKAAAAKAAAKA-CONH_2_), which was subsequently subjected to iterative computational sequence optimization. The 3D structure of the helical peptides was generated using the Discovery Studio Visualizer tool. Subsequently, one of the rationally designed peptides (SR18) was custom-synthesized using the standard Fmoc (Fluorenylmethoxycarbonyl protecting group) solid-phase peptide synthesis method by availing the commercial services of GenScript Biotech. Post cleavage from the resin, the peptide was purified (≥95%) to homogeneity. The analytical high-performance liquid chromatography (HPLC) profile of the peptide was recorded by using a C18 (4.6 × 250 mm^2^) column at 220 nm under an acetonitrile-water gradient in the presence of 0.05%–0.065% trifluoroacetic acid (TFA). Electrospray ionization mass spectrometry (ESI-MS) confirmed the integrity of the peptide.

### 2.4 Circular dichroism (CD) studies

The far-UV circular dichroism (CD) spectra of the SR18 were recorded in a quartz (Helma) cell with a path length of 0.1 cm at 25 °C under different solvent conditions, such as 1X PBS (phosphate buffered saline), 30 mM SDS (Sodium dodecyl sulphate), 20% TFE (2,2,2-trifluroethanol), and 20% HFIP (1,1,1,3,3,3-hexafluro-2-propanol), by using the Chirascan CD spectrometer system (Applied Photophysics). The CD spectra were recorded with a time constant of 1 s and a step size of 1 nm, averaging three scans. The peptide stock solution was diluted in the respective solvents, and the peptide concentration was maintained at 100 μM by monitoring the absorbance at 280 nm. After background subtraction of corresponding solvents, the observed ellipticity in millidegrees was converted to mean residual ellipticity [θ_MRE_, degcm^2^·dmol^-1^] using the following equation (millidegrees × mean residual weight)/(path length in millimeters × concentration in mg·mL^-1^) (Greenfield, 2006). The ⍺-helix fraction (*f*_*a*_) of SR18 at *θ*_222_ was calculated as the ratio of [*θ*_222_]experimental to [*θ*_222_]theoretical, as described here (Hamley et al., 2025). All data points were plotted in GraphPad Prism, and the spectra were smoothed up to 2–3 units wherever required.

### 2.5 Dynamic Light Scattering (DLS) studies

SR18 dissolved in filtered 1X PBS (pH ∼ 7.4) and 20% TFE to a final concentration of 100 μM was subjected to DLS measurement to gauge its hydrodynamic size and particle size distribution, using bovine serum albumin as the reference. Prior to the DLS measurement, the sample was filtered through a 0.22 μm filter to eliminate the dust particles that could interfere with the measurement. The analysis was performed at 25 °C using a Zetasizer Nano ZS (Malvern Instruments) equipped with a 633 nm He-Ne laser, with scattered light detected at 90°. Each measurement consisted of three independent runs, each averaging five acquisitions.

### 2.6 Cytotoxicity studies

The cytotoxicity profile of SR18 was examined using the 3-(4,5-dimethylthiazol-2-yl) 2,5-diphenyltetrazolium bromide (MTT) assay utilizing HEK-293 and HeLa cell lines obtained from the School of Biological Sciences, NISER, Bhubaneswar. Approximately 4 × 10^4^ cells per well were inoculated into 96-well plates containing Dulbecco’s modified Eagle’s medium (DMEM) enriched with 10% fetal bovine serum and penicillin-streptomycin (10 mL/L), and incubated for 24 h at 37 °C with 5% CO_2_. Thereafter, the cells were treated with serially diluted SR18 in culture media and incubated for an additional 18 h at 37 °C in 5% CO_2_. Subsequently, 10 µL of MTT solution (5 mg/mL) was introduced to each well, and the plates were incubated for a further 4 h. After 4 h, the suspensions were removed, and 100 µL of dimethyl sulfoxide (DMSO) was added to dissolve the formazan crystals. The plates were further incubated at 37 °C with 5% CO_2_ for 30 min to achieve complete solubilization. The absorbance was measured at 570 nm using a SpectraMax iD3 microplate reader (Molecular Devices). Cells treated with PBS served as the negative control, whereas cells exposed to 10% DMSO served as the positive control. After subtracting blank absorbance (medium only), cell viability (%) was calculated as the ratio of OD_570_ of treated cells to OD_570_ of untreated cells. To ensure reproducibility, all experiments were conducted in triplicate and repeated three times independently (n = 3).

### 2.7 Hemolytic Assay

Approximately 2 mL of human blood was collected from an anonymous healthy volunteer at the Sanjeevan Health Center, Indian Institute of Technology Bhubaneswar, following written informed consent. Blood was collected in a K2-EDTA-coated tube to prevent coagulation. Erythrocytes were separated by centrifugation at 2,500 rpm for 10 min at room temperature. The collected erythrocytes were then washed four to five times with sterile 1X PBS, with each wash including 10 min of centrifugation, until the supernatant was clear. The purified erythrocytes were then diluted in 1X PBS to a final ratio of 1:50 (Erythrocytes: 1X PBS). Aliquots of the diluted erythrocyte suspension were introduced into microcentrifuge tubes containing 10 μL of SR18 with concentrations ranging from 0.39 to 100 μM, resulting in a final reaction volume of 100 μL. The mixtures were incubated at 37 °C for 60 min, then centrifuged at 2,500 rpm for 10 min at room temperature. After that, ∼50 μL of supernatant was carefully transferred to microtiter plate wells without disturbing the cell pellet, and the cell lysis of the erythrocytes was quantified by measuring OD at 414 nm using SpectraMax iD3 microplate reader (Molecular Devices). Cells treated with 10 μL of 10% Triton X-100 served as the positive control, while cells treated with 10 μL of sterile PBS served as the negative control. All experiments were carried out in triplicate on two distinct experimental sets (n = 2). The proportion of hemolysis was estimated using the formula (Abs_peptide_-Abs_PBS_)/(Abs_triton_-Abs_PBS_) × 100.

### 2.8 Proteolysis studies

SR18 was incubated with trypsin at an enzyme-to-peptide ratio of 1:20 (trypsin: peptide) in 50 mM Tris−HCl (pH ∼ 8) at 37 °C with moderate agitation for ∼4 h to evaluate its susceptibility to proteolytic degradation. Subsequently, the reaction mixture was heated to 100 °C for 30 min to inactivate trypsin, then centrifuged at 13,000 g for 30 min to precipitate the enzyme. The resultant supernatant was collected and analyzed using an Exactive Plus Orbitrap liquid chromatography-high-resolution mass spectrometry (LC-HRMS) instrument from Thermo Scientific.

### 2.9 Molecular dynamics studies

The GLP-1R, respectively complexed to GLP-1 and SR18, was embedded into a 75% POPC (1-palmitoyl-2-oleoyl-sn-glycero-3-phosphocholine) and 25% cholesterol (CHL) bilayer using CHARMM-GUI (Jo et al., 2008) before MD simulation. The GLP-1−GLP-1R and SR18−GLP-1R complexes were subjected to MD simulations of 1 μs at 300 K in TIP3 water using CHARMM36 m forcefield (Huang et al., 2017) parameters in the GROMACS engine (Hess et al., 2008), as described in our previous studies (Gupta et al., 2025). Before the production MD run, both systems were energy-minimized to a tolerance of 1,000 kJ mol^-1^nm^-1^ using the steepest descent method, first in a vacuum and then in the presence of TIP3 water, with a solvent density set to the value corresponding to 1 atm at 300 K. Furthermore, the net charge of the system was neutralized by randomly placing the required number of counterions in the box to maintain a NaCl concentration of 0.15 M. The final GLP-1−GLP-1R complex contained 72,067 atoms, including 16,320 TIP3 water, 44 sodium, 99 chloride, 102 POPC, and 33 CHL molecules, whereas the SR18−GLP-1R complex contained 79,689 atoms, including 18,915 TIP3P water, 51 sodium, 54 chloride, 102 POPC, and 33 CHL molecules. Both systems were equilibrated twice, first under constant number, volume, and temperature (NVT: 5 ns) conditions, and then under constant number, pressure, and temperature (NPT: 10 ns) conditions before the start of the production MD run. With a coupling time constant of 1 ps, the protein and solvent were coupled independently to a V-rescale bath at 300 K. H-bonds were constrained with LINCS with order 4. The non-bonded pair list cutoff was 1.2 nm with a grid function. Numerical integrations were performed in steps of 2 fs, and the coordinates were updated every 10 ps. Conformational clustering of both the trajectories (SR18−GLP-1R and GLP-1−GLP-1R) was performed every 50 ps with a root mean square deviation (RMSD) cutoff of ≤2.5 Å by recruiting the GROMOS fitting method, as defined in GROMACS. The MD trajectories were thoroughly analyzed by recruiting the utility modules available in GROMACS. A similar process was followed for the MD studies of GLP-1, Semaglutide, and SR18 in the presence of TIP3 water. The MD trajectories of the peptides were subjected to conformational clustering at 50 ps intervals using an RMSD cutoff of ≤1.5 Å.

### 2.10 Estimation of the free energy of binding

The following equation, ΔG_binding_ = G_complex_ −(G_protein_ + G_ligand_), implemented in the gmx_mmpbsa program (Valdes-Tresanco et al., 2021), was used to calculate the binding free energy of the complexes using the Molecular Mechanics Poisson-Boltzmann surface area (MM-PBSA) method. The following equation estimated the free energy contribution: G = E_MM_ + G_(solv)_ – TS_(solute)_, where E_MM_ (molecular mechanics energy) represents the summation of van der Waals and electrostatic, G_(solv)_ represents the solvation energy contributed by both polar and non-polar solvation free energy, and TS_(solute)_ represents the temperature and entropy of the solute. Finally, 300 structures, including 200 conformers randomly selected from the first major cluster and 100 from the second major cluster, were used from the respective MD trajectories to calculate the total binding free energy and decomposition energy of the complexes.

## 3 Results

### 3.1 Molecular dynamics (MD) simulation of GLP-1 complexed to loop remodeled GLP-1R

**FIGURE 3:**
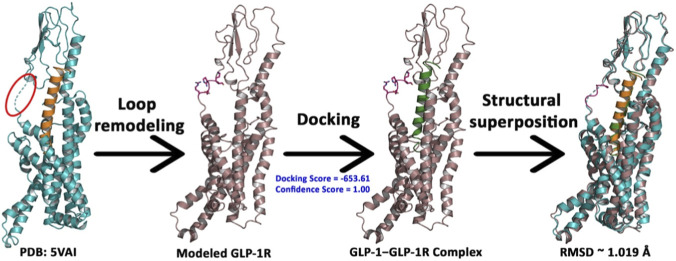
Docking of native GLP-1 (green) to the loop-remodeled GLP-1R with structural superposition against the cryo-EM reported structure of GLP-1 activated GLP-1R (PDB: 5VAI). The amino acid side chains of the remodeled loop region are shown in pink sticks.

The initial structure of GLP-1R was retrieved from the reported active structural complex of GLP-1R with the native GLP-1 (PDB: 5VAI). The unresolved loop residues (S^129^RRGEK^134^) connecting the N-terminus to the transmembrane helix 1 (TM1) of GLP-1R were appropriately remodeled using MODELLER. The native GLP-1 was then docked to the loop-remodeled GLP-1R using the HDOCK web server, which generated a GLP-1R complex with GLP-1 with a docking energy score of −653.61 and a confidence score of 1. The structural superposition of the modeled complex with 5VAI indicated a backbone RMSD of 1.079 Å (Figure 3), suggesting that the model complex is suitable for further studies.

**FIGURE 4:**
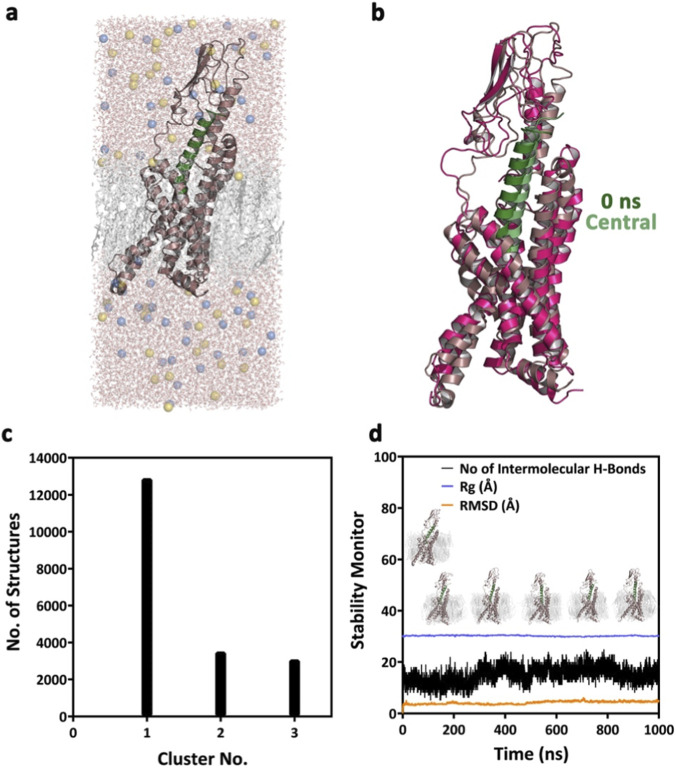
**(a)** The energy-minimized model complex of GLP-1–GLP-1R, embedded in the model POPC + CHL bilayer. **(b)** Structural superposition (RMSD: 3.057 Å) of the GLP-1–GLP-1R complex with the central conformer of the most populated cluster of the complex, as evolved over 1 μs of MD simulations. **(c)** Major conformational clusters of the GLP-1–GLP-1R complex evolved over 1 μs of MD simulations. **(d)** The overall structural stability of the GLP-1–GLP-1R complex over the duration of the MD simulations.

The model GLP-1–GLP-1R complex was embedded in a POPC-CHL (75:25) bilayer using CHARMM-GUI and subsequently subjected to MD simulations for 1 μs at 300 K as a positive control. The MD data presented in Figure 4 suggest that the GLP-1–GLP-1R complex remains structurally stable throughout the MD simulations, with an average of 15 intermolecular hydrogen bonds between GLP-1 and GLP-1R. Further analysis of the MD trajectory reveals that the central conformer of the most populated cluster of the GLP-1−GLP-1R complex evolved over 1 μs of MD simulations, shares an RMSD of 3.057 Å (Figure 4) with the starting model complex. Notably, the remodeled section of the loop, which was originally not resolved in the cryo-EM studies, adopted an altogether different conformation in the most populated conformer of the complex.

**FIGURE 5:**
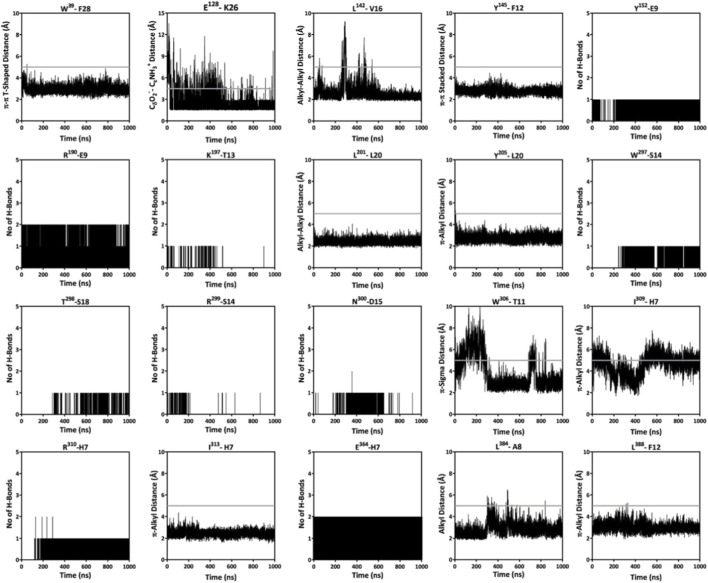
Monitoring the sustainability of intermolecular (hydrogen, electrostatic, and hydrophobic) interactions observed between GLP-1 and GLP-1R throughout the MD simulation. The GLP-1R residues are shown in superscript. The grey line highlights the cutoff distance for the specified interaction between the amino acids.

Further analysis of the trajectory suggests that the several important amino acids, such as H7, E9, T11, F12, D15, and F28 of GLP-1 actively interact with the W^39^, Y^145^, Y^152^, R^190^, K^197^, L^201^, N^300^, R^310^, E^364^, L^384^, and L^388^ of GLP-1R through several hydrogen, hydrophobic, and electrostatic interactions (Figure 5; Supplementary Figure S1). Interestingly, H7, E9, and D15 of GLP-1 destabilize TM6 and ECL3, thereby facilitating the GLP-1R in attaining its active conformation. Further, the H7 of GLP-1 interacts with TM3 and TM5 through hydrogen and hydrophobic interactions with R^310^, I^313^, and E^364^ of GLP-1R. Notably, earlier alanine substitution studies have revealed that Y^152^, R^190^, K^197^, L^201^, *R* ^299^, N^300^, W^306^, R^310^, I^313^, E^364^, L^384^, L^388^ of GLP-1R are the most important residues for GLP-1 binding affinity and potency. Interestingly, except for Y^148^, D^198^, M^233^, Q^234^, Y^235^, W^284^, D^372^, E^373^, R^380^, K^383^, and E^387^, all other residues were found to maintain sustained intermolecular interactions with GLP-1 over the duration of the MD simulations (Dods and Donnelly, 2015; Liang et al., 2018; Zhang et al., 2020; Deganutti et al., 2022). Therefore, it is reasonable to assume that GLP-1 analogs that could establish stable interaction with these residues of GLP-1R can produce therapeutic effects similar to the current GLP-1R targeting peptides for the management of T2DM.

### 3.2 Rational design of an α-helical peptide agonist for targeting GLP-1R

Sequence comparison of GLP-1 (30 aa) with the other GLP-1 mimetics, such as Taspoglutide (30 aa), Liraglutide (31 aa), and Semaglutide (31 aa), indicates that strategic incorporation of NCAAs, such as Aib, and chemical modification of specific Lys side chains have been successful in improving the pharmacokinetics of the GLP-1 mimetics without affecting their interactions with GLP-1R compared to the native GLP-1. Further, analysis of the reported cryo-EM data of GLP-1–GLP-1R complex indicates that certain residues on GLP-1 do not make significant contact with the ECD of the GLP-1R (Zhang et al., 2017). Therefore, for rational design of peptide mimetics, the entire sequence of GLP-1 may be divided into the N-terminal (14 aa), connector (5 aa), and C-terminal (11 aa) regions, which are collectively important for binding and signaling of GLP-1R. Since short peptides ranging from nine to eleven amino acids have been discussed in the literature earlier as potent GLP-1RAs, we decided to design an intermediate-sized potent peptide (≤20 aa) agonist of GLP-1R by conserving several critical residues of GLP-1 important for GLP-1R binding and signaling. Notably, in our earlier studies, we demonstrated the impact of a few NCAAs on the conformational stability of model helical peptides composed of ≤18 amino acids (Behera et al., 2022), which we subsequently subjected to sequence optimization, producing a potent antimicrobial peptide (Behera et al., 2024a) and a potential antiviral peptide (Behera et al., 2024b) binder targeting the receptor binding domain of SARS-CoV-2.

**FIGURE 6:**
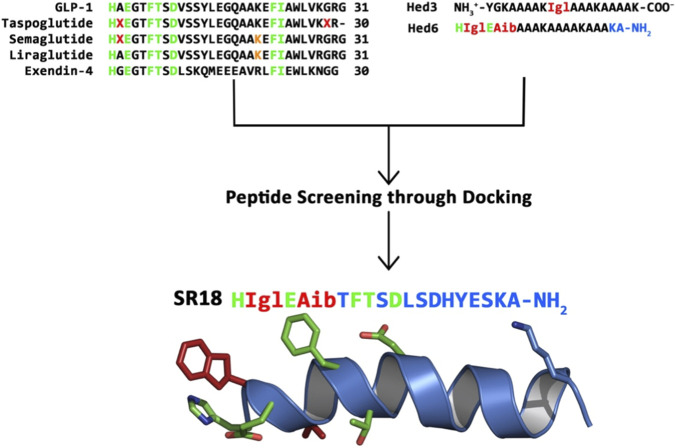
Schematic illustration of the sequence optimization process utilized in the rational design and selection of SR18. Residues highlighted in green represent the critical conserved residues. The red-colored amino acids represent NCAAs (Igl, X = Aib). The lysine residue, which is chemically modified in GLP-1 analogs, is highlighted in orange.

Therefore, in the current study, we utilized one of the model helical peptides (Hed3: NH_3_^+^-YGKAAAAK-Igl-AAAKAAAAK-CO_2_^−^) to produce the template GLP-1 mimetic peptide (Hed6: NH_3_^+^-H-Igl-E-Aib-AAAKAAAAKAAAKA-CONH_2_) and further subjected it to iterative computational sequence optimization to produce a potent helical peptide agonist (SR18) of GLP-1R (Figure 6). The Lys residue at the 17th position was introduced at the C-terminus with an objective to modify its sidechain with a linker in the future (Supplementary Figure S2), so that the peptide can anchor to serum albumin similar to Semaglutide (Knudsen and Lau, 2019). Additionally, the C-terminus was amidated to protect the peptide from carboxypeptidase degradation.

The first four residues of the N-terminus were also fixed for the following reasons. His1 and Glu3 were fixed as critical residues for GLP-1R (Adelhorst et al., 1994; Xiao et al., 2001) Further, whereas Igl (Indanyl glycine), a glycine derivative, was introduced at the second position to protect the dipeptide cleavage by DPP-4 and to improve metabolic stability, including imparting necessary conformational constraints to maintain an appreciable binding affinity at the active site of GLP-1R, similar to the small-molecule GLP-1RAs. Furthermore, the Aib at the fourth position was fixed to help the peptide induce a helical turn at the N-terminus (Karle and Balaram, 1990). Notably, Aib has been incorporated into GLP-1RAs, such as Taspoglutide, Semaglutide, and Tirzepatide, to improve their therapeutic index. The remaining amino acid positions were iterated to produce ∼200 unique peptides, each composed of ≤18 amino acids, out of which 41 peptides were initially shortlisted and ranked (Supplementary Table S1) through screening against loop-remodeled GLP-1R bound to GLP-1 based on their binding energy score and orientation using the HDOCK webserver.

**FIGURE 7:**
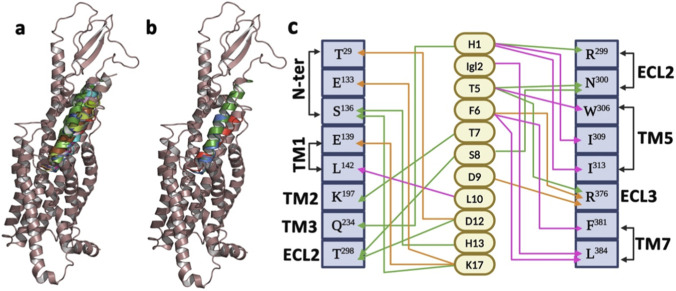
**(a)** Structural superposition of the designer peptides in the context of GLP-1 bound to the modeled GLP-1R. **(b)** Comparison of the binding orientation of the top-ranked peptide (red, binding score: −352.98) with the SR18 (blue, binding score: −272.14) in the context of GLP-1 (green, binding score: −653.61). The SR18 demonstrates a backbone RMSD of 0.948 Å with GLP-1, whereas the top-ranked peptide demonstrates a backbone RMSD of 1.094 Å with GLP-1 and an RMSD of 0.721 Å with SR18. **(c)** Illustration of various intermolecular interactions observed in the docking pose between SR18 and GLP-1R. The green line indicates hydrogen bonding, the pink line indicates hydrophobic interactions, and the orange line indicates electrostatic interactions.

The binding energy scores of the peptides ranged between −352.98 (top rank, confidence score: 0.98) and −202.07 (bottom rank, confidence score: 0.73), compared to GLP-1 (−653.61, confidence score: 1.00), with higher negative values indicating relatively stronger binding affinity. Furthermore, a confidence score >0.7 indicates a higher likelihood of binding to the receptor. Subsequently, 22 peptides (∼50% of the shortlisted peptides) with a confidence score ≥0.92 were superimposed in the context of GLP-1 bound to GLP-1R (Figure 7). Notably, the docking orientation of the top-ranked peptide was observed to be different compared to that of the GLP-1. Interestingly, exendin-4 (9–39), an antagonist of GLP-1R (Avexitide, Phase III), has been shown earlier to bind to the ECD of GLP-1R with an orientation different than that of GLP-1. Notably, activation of GLP-1R requires specific coordinated interactions with potential ligands, as observed in GLP-1 and Semaglutide. Therefore, all high-affinity binding ligands may not necessarily behave as GLP-1RAs and may potentially exhibit alternative functions, such as biased agonists, inverse agonists, and antagonists. In fact, it is reported (Underwood et al., 2010) that exendin-4 (9–39) binds strongly (EC_50_ ∼5 pM, IC_50_ ∼0.76 nM) to wild-type GLP-1R than the native GLP-1 (EC_50_ ∼11 pM, IC_50_ ∼1 nM). Moreover, the top-ranked peptide did not contain the amino acids in its sequence considered important for activating the GLP-1R and was thus reserved for future evaluations.

However, another peptide, codenamed SR18 (H-Igl-E-Aib-TFTSDLSDHYESKA-CONH_2_), which contained several amino acids similar to those found on the GLP-1 and demonstrated a docking orientation similar to the GLP-1 with a binding energy score of −272.14, was rationally shortlisted for further studies as a first-generation GLP-1RA over the top-ranked peptide (H-Igl-E-Aib-AWMTSIISTMWAKH-CONH_2_). Interestingly, SR18 also docked to the most populated conformer of the loop-remodeled GLP-1R bound to GLP-1, evolved over 1 μs of the MD simulations in a POPC-CHL bilayer. Notably, SR18 occupied the same orthosteric binding pocket on both conformers of GLP-1R, a binding site typically occupied by GLP-1 and other similar peptide mimetics, as evidenced in reported structural studies (Zhang et al., 2017; Zhang et al., 2020). A comparative docking energy (confidence score) and binding energy for SR18 in reference to GLP-1, Semaglutide, and Avexitide are provided in Table 1.

**TABLE 1:** A comparative docking (binding) energy score of SR18 in reference to GLP-1 and Semaglutide against different conformers of GLP-1R.

Peptide	Receptor	HDOCK energy (confidence) score	PRODIGY energy (K_d_)
GLP-1	GLP-1R (0 ns)	−653.61 (1.00)	−12.9 kcal/mol (0.49 nM)
	GLP-1R (central conformer)	−328.81 (0.97)	−12.2 kcal/mol (1.2 nM)
Semaglutide	GLP-1R (0 ns)	−478.56 (0.99)	−12.8 kcal/mol (0.44 nM)^a^
​	GLP-1R (central conformer)	−448.09 (0.99)	−13.7 kcal/mol (0.09 nM)^a^
SR18	GLP-1R (0 ns)	−272.14 (0.92)	−9.9 kcal/mol (55 nM)^a^
	GLP-1R (central conformer)	−237.30 (0.85)	−8.8 kcal/mol (360 nM)^a^
Avexitide	GLP-1R (0 ns)	−330.94 (0.97)	−10.7 kcal/mol (14 nM)^a^
	GLP-1R (central conformer)	−294.52 (0.94)	−10.3 kcal/mol (29 nM)^a^

^a^ Calculated by mutating Aib to Ala and Igl to Phe, respectively, in Semaglutide and SR18. SeMet was mutated to Met in Avexitide.

### 3.3 Evaluation of conformational stability of SR18

Notably, SR18 is an 18-amino acid designer polypeptide, modeled as a helix composed of both coded and NCAAs. On the contrary, both GLP-1, the native agonist of GLP-1R, and Semaglutide, the FDA-approved GLP-1 analog, are 31-amino acid helical polypeptides. However, Semaglutide shares ∼94% sequence homology with GLP-1 and harbors a single NCAA (Aib2) and a fatty acid-modified Lysine (Lys20) in its sequence. Therefore, in reference to GLP-1 and Semaglutide, it was pertinent to evaluate whether SR18 in its unbound state could sustain the helical conformation in solution. Thus, for comparison, both GLP-1 and SR18 were submitted to MD simulations for over 100 ns in explicit water at 300 K, including the deacylated Semaglutide.

**FIGURE 8:**
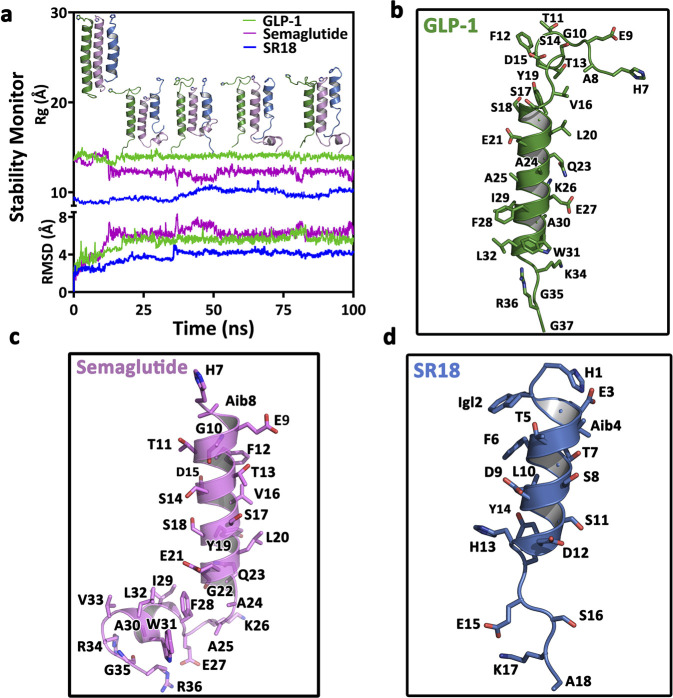
**(a)** Comparison of conformational stability of SR18 in reference to GLP-1 over 100 ns MD simulation at 300 k **(b)** The central conformer of the GLP-1 of the major cluster evolved in MD studies. **(c)** The central conformer of Semaglutide from the first major cluster evolved over the MD studies. **(d)** The central conformer of SR18 in the major cluster evolved over the MD studies.

The comparative data presented in Figure 8 (Supplementary Figure S3) suggest that SR18 can predominantly sustain a helical conformation in solution, similar to GLP-1 and Semaglutide, with some noticeable end fraying, specifically in the C-terminus region, which is generally observed for short peptides. In contrast, end fraying was observed at the N- and C-termini of GLP-1. On the other hand, the deacylated Semaglutide maintained its helical structure with some minimal end fraying observed in the C-terminus region.

### 3.4 Evaluation of basic pharmacological profile of SR18

**FIGURE 9:**
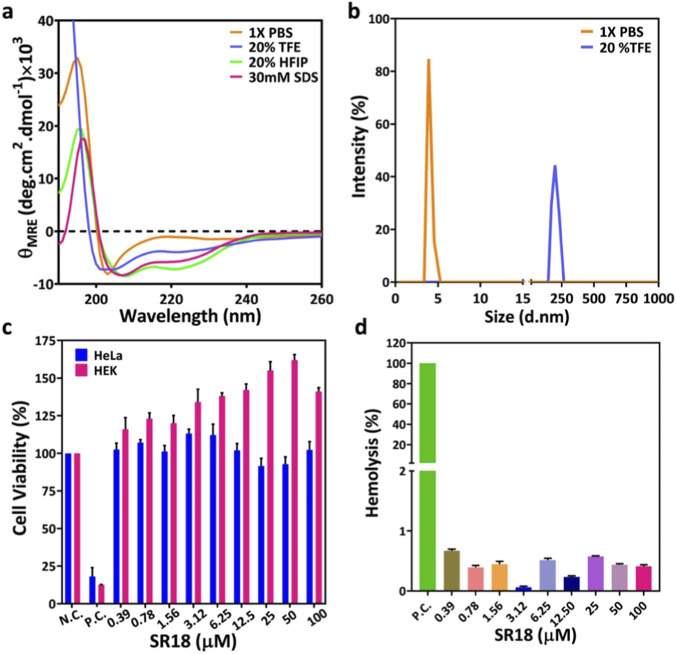
**(a)** The far-UV CD signature of 100 μM SR18 recorded under different solvent conditions at 25 °C. **(b)** The hydrodynamic size distribution of 100 μM SR18 in 1X PBS and 20% TFE. **(c)** The cytotoxicity profile of SR18 in HeLa and HEK293 cells across a concentration range of 0.39–100 μM, respectively, using 1X PBS as a negative control (NC), and 10% DMSO as a positive control (PC). **(d)** The hemolytic profile of SR18 against freshly isolated human erythrocytes across a concentration range of 0.39–100 µM using 10% Triton-X as PC.

Taking cue from the comparative MD simulation data, SR18 was custom-synthesized and purified to homogeneity, with a purity level of ≥95%, as evident from the analytical HPLC profile. The ESI-MS data further confirmed the integrity of SR18. The monoisotopic mass (O_Mol.Wt_: 2,124.0) was found to be consistent with the theoretical molecular weight (T_Mol.Wt_:2,124.32) of the peptide (Supplementary Figure S4). Interestingly, the CD spectra of the SR18 also demonstrated a signature signal in the far-UV region around 208 and 222 nm, typically observed for moderately ordered short ⍺-helical peptides. Notably, the ⍺-helical signal of SR18 improved further in the presence of the cosolvents, further confirming its ability to adopt a helical conformation under various solvent/cosolvent conditions, including PBS, 20% HFIP, 20% TFE, and 30 mM SDS (Figure 9a). Further, the ⍺-helix fractions (*f*_*a*_) of SR18 calculated at *θ*_222_ were found to be in the range of 0.034 in 1X PBS, 0.12 in 20% TFE, 0.22 in 20% HFIP, and 0.17 in 30 mM SDS (Supplementary Table S2), which is noted to be comparable with the reported value of 0.027 for semaglutide (Hamley et al., 2025).

Further, Dynamic Light Scattering (DLS), a widely used technique for determining the hydrodynamic size and size distribution of molecules in a sample by analyzing fluctuations in scattered light due to Brownian motion, was used to evaluate the hydrodynamic size distribution of 100 μM SR18 under different solvent conditions. DLS measurement was carried out in 1X PBS (pH ∼ 7.4) and 20% TFE at 25 °C (Figure 9b). SR18 exhibited an average hydrodynamic diameter of 4.001 nm with a polydispersity index (PDI) of 0.2289, indicating a relatively uniform and predominantly monomeric population in 1 X PBS, compared to 10 μM BSA (hydrodynamic diameter 7.631 nm and PDI 0.1645) as shown in Supplementary Figure S5. In contrast, in 20% TFE, SR18 displayed a significantly higher hydrodynamic diameter of 230.7 nm with a PDI of 0.4292, suggesting that SR18 may form higher-order aggregates or self-assembled structures. Notably, depending on the formulation, Semaglutide is reported to have a particle size ranging from ∼4 to 3,000 nm in DLS measurements (Li et al., 2024; Pinto et al., 2024; Hamley et al., 2025; Lee et al., 2025).

Cytotoxicity profiling of designer peptides is crucial to ensure that they are generally safe and do not harm healthy cells while maintaining therapeutic efficacy. Thus, the cytotoxicity profile of SR18 was evaluated by recruiting HeLa and HEK293 cell lines. As noted in Figure 9c, the viability of HeLa or HEK cells was not substantially impacted by SR18 at concentrations ranging from 0.39 to 100 µM when compared to cells that were treated with 1X PBS alone. In contrast, under the same experimental conditions, 10% DMSO exhibited pronounced cytotoxicity towards both HeLa and HEK293 cell lines, due to its ability to induce cellular stress by developing reactive oxygen species. Thus, the data primarily suggest that SR18 is non-toxic to eukaryotic cells, even at a high concentration under the cell culture conditions.

Similarly, the hemolytic profile of the designer peptides is also essential for assessing their biocompatibility and selectivity towards human erythrocytes. Thus, the hemolytic profile of SR18 was evaluated using a standard hemolytic assay by incubating freshly isolated human erythrocytes with SR18 at concentrations ranging from 0.39 to 100 µM. As judged from the absorbance of hemoglobin at 414 nm shown in Figure 9d, SR18 does not induce significant hemolysis of human RBCs, even at the highest concentration tested. In contrast, 10% Triton X-100, used as a positive control, caused pronounced erythrocyte hemolysis. Thus, these results demonstrate that SR18 is non-hemolytic and non-toxic to human erythrocytes.

**FIGURE 10:**
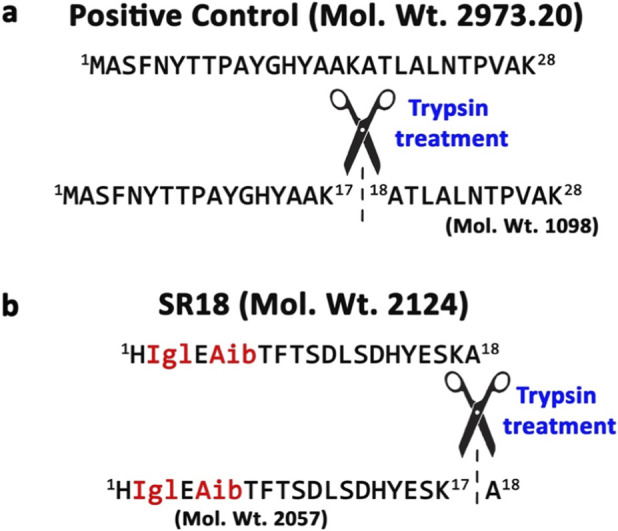
Schematic illustration of the trypsin cleavage sites, respectively, on the **(a)** positive control SR4, and **(b)** SR18. The NCAAs are indicated in red.

Additionally, proteolytic degradation is a major challenge in peptide-based therapeutics, as it results in short half-life, low bioavailability, rapid clearance, and ultimately reduced therapeutic efficacy. This instability often necessitates frequent dosing and can lead to unfavorable pharmacokinetic profiles and potential immunogenic responses from degraded fragments. Though SR18 was designed to resist proteolysis by DPP4 due to the presence of Igl2, the inclusion of Lys17 for future acylation could make SR18 susceptible to proteolysis by other enzymes. Therefore, SR18 was incubated with trypsin (trypsin:peptide = 1:20) for ∼4 h, using SR4, another peptide reported earlier (Ghosh et al., 2024) from our lab as a positive control (Figure 10). The presence of a peak at ∼1,098 Da in the HRMS data (Supplementary Figure S6) corresponding to the fragment expected from cleavage of the amide bond between Lys17 and Ala18 further confirms trypsin’s enzymatic efficiency, as its activity depends on pH, temperature, and peptide structure.

Notably, SR18 also contains a potential trypsin cleavage site (Figure 10), and the HRMS data (Supplementary Figure S7) with a peak around ∼685 Da confirm that trypsin can cleave SR18 between Lys17 and Ala18, leaving the remaining crucial 17 residues intact. Thus, SR18 is likely to retain its function even if partial cleavage occurs under *in vivo* conditions. Additionally, Lys17, upon further modification (e.g., K26 in Semaglutide), is expected to enhance the binding affinity of SR18 to serum albumin (Supplementary Figure S2), improving its *in vivo* pharmacological functions. In summary, SR18 has the desired basic pharmacological profile; however, establishing it as a potential lead peptide agonist of GLP-1R requires further experimental evaluations. Although cell signaling studies are desirable for further evaluation of SR18 for obvious reasons, as an alternative, the agonistic potential of SR18 was preliminarily evaluated using computational structural biology techniques instead. In the first phase of the current study, SR18, docked to GLP-1R, was subjected to 1 μs MD simulations to directly compare its agonist-like properties with the established agonists and antagonists of GLP-1R reported earlier.

### 3.5 Molecular dynamics (MD) simulation of SR18 complexed to loop remodeled GLP-1R

**FIGURE 11:**
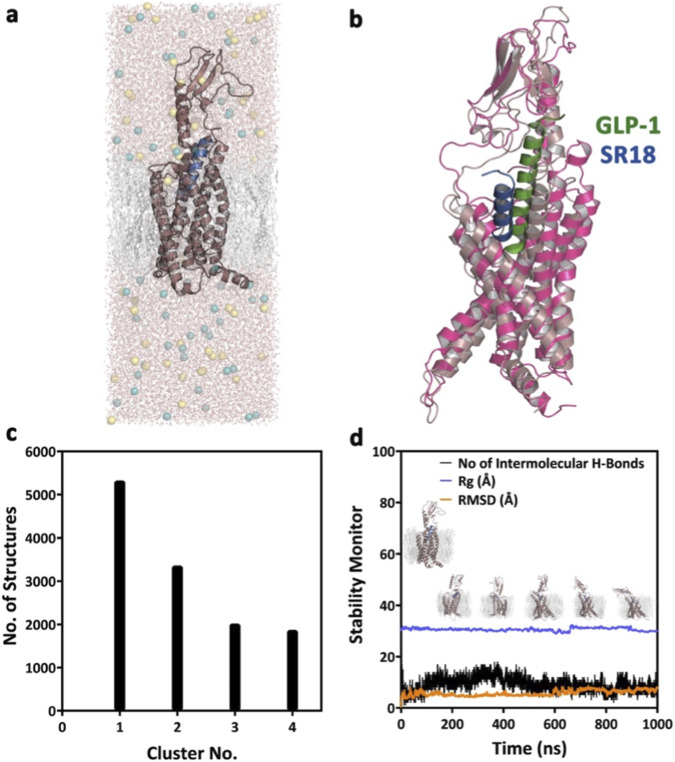
**(a)** The energy-minimized model complex of SR18−GLP-1R, embedded in the model POPC + CHL bilayer. **(b)** Structural superposition (RMSD: 3.287 Å) of the SR18–GLP-1R complex with the central conformer of the most populated cluster of the complex, as evolved over 1 μs of MD simulations. **(c)** Major conformational clusters of the SR18−GLP-1R complex evolved over 1 μs of MD simulations. **(d)** The overall structural stability of the SR18–GLP-1R complex over the duration of the MD simulations.

The SR18–GLP-1R complex embedded in a POPC-CHL (75:25) bilayer was subjected to MD simulation studies over 1 μs at 300 K, similar to the GLP-1–GLP-1R complex. The trajectory analysis of the SR18−GLP-1R complex (Figure 11) revealed that, similar to GLP-1, SR18 also remains bound to the GLP-1R throughout the MD studies with an average of nine intermolecular hydrogen bonds.

**FIGURE 12:**
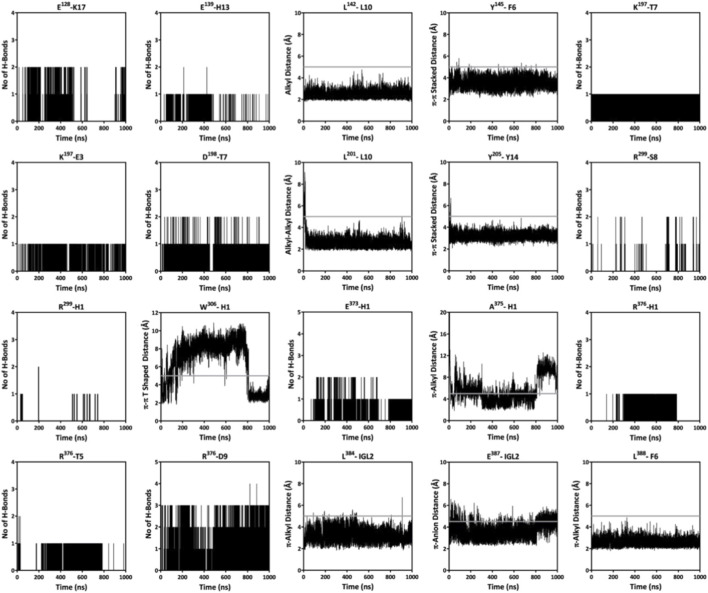
Monitoring the sustainability of intermolecular (hydrogen, electrostatic, and hydrophobic) interactions observed between SR18 and GLP-1R throughout the MD simulation. The GLP-1R residues are shown in superscript. The grey line highlights the cutoff distance for the specified interaction between the amino acids.

Further, like GLP-1, the C-terminal amino acids of SR18 also interact with the N-terminal region of GLP-1R. Additionally, like GLP-1, SR18 also engages with the residues of TM1, TM2, TM6, and TM7 of GLP-1R. Notably, H1, Igl2, E3, F6, T7, S8, L10, D12, and Y14 of SR18 demonstrate similar interaction patterns with K^197^, L^201^, *R* ^299^, W^306^, L^384^, and L^388^ of GLP-1R. Additionally, SR18 also interacts with other important residues of GLP-1R, including D^198^, E^373^, and E^387^. The common GLP-1R residues that interact with both peptides and maintain stable intermolecular interactions throughout MD simulations are presented through the interaction map in Figure 12 (Supplementary Figure S8). Interestingly, these residues of the GLP-1R are crucial for intracellular signaling and are responsible for insulin exocytosis.

### 3.6 Comparative estimation of free energy of binding between SR18−GLP-1R and GLP-1−GLP-1R complexes

**FIGURE 13:**
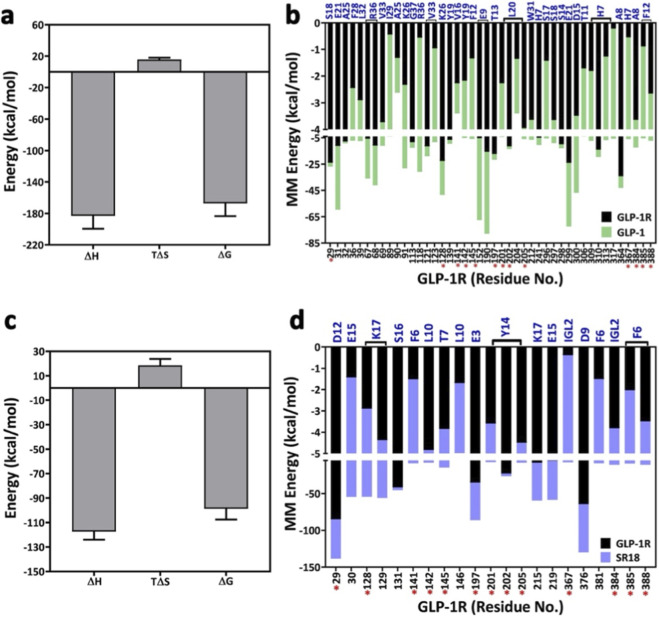
**(a)** Summary of the total binding free energy calculations for GLP-1−GLP-1R complex. **(b)** Residue-specific energetic contribution of GLP-1 toward binding of GLP-1R. **(c)** Summary of the total binding free energy calculations for SR18−GLP-1R complex. **(d)** Residue-specific energetic contribution of SR18 toward the binding of GLP-1R. The red asterisks represent the GLP-1R residues common to both GLP-1 and SR18.

To further understand the energetic contribution of the crucial residues of GLP-1R toward SR18 compared to GLP-1, the MD trajectories were subjected to gmx_MMPBSA calculations. For the calculation, 300 structures were randomly extracted from the first two major clusters of both complexes. The data in Figure 13 summarizes the energetic contributions of GLP-1R residues, along with critical, or more precisely, the “hotspot” amino acids that contribute to GLP-1R activation and insulin exocytosis. Given the relatively small size of SR18, the estimated binding free energy for SR18−GLP-1R complex is noted to be −98.79 ± 8.77 kcal/mol compared to −167.22 ± 16.20 kcal/mol observed for GLP-1−GLP-1R complex, which suggests that SR18 strongly binds to GLP-1R. Further decomposition analysis of the data reveals that the energetic contribution of 13 important residue pairs is conserved between the SR18−GLP-1R and GLP-1−GLP-1R complexes. Therefore, it is reasonable to believe that SR18 can potentially act as an agonist of GLP-1R.

## 4 Discussions

**FIGURE 14:**
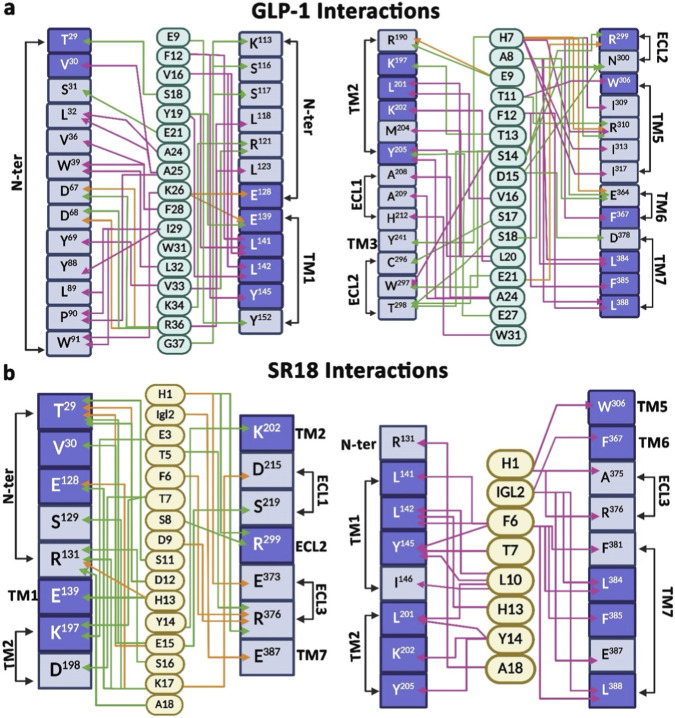
**(a)** The interaction map of GLP-1 with GLP-1R. **(b)** The interaction map of SR18 with GLP-1R. The common residues of GLP-1R that interact with SR18 and GLP-1 are highlighted in purple shades. The green line indicates hydrogen bonding, the pink line indicates hydrophobic interactions, and the orange line indicates electrostatic interactions.

Over the years following its discovery, GLP-1 has garnered significant attention for its incretin effect on glucoregulation in the management of T2DM, including its role in improving cardiovascular, respiratory, and renal functions (Avogaro and Fadini, 2025). GLP-1 is known to induce a reduction in caloric intake by altering appetite, satiety, and prospective food consumption (Astrup, 2024; Bettadapura et al., 2025). Notably, chronic hyperglycemia can trigger neuroinflammation, and interestingly, GLP-1 can elicit neuroprotection by producing an anti-inflammatory effect through the recruitment of GLP-1R in the central nervous system (Diz-Chaves et al., 2024; Singh et al., 2025b). Interestingly, in the absence of experimental data, ADMETlab 3.0 (Fu et al., 2024) prediction suggests that SR18 has excellent blood-brain barrier (BBB) permeability (prediction probability value: 0–0.1), identical to that of GLP-1. However, GLP-1 has a short half-life, which provides the desired impetus to develop analog compounds that can mimic its physiological functions. In addition to secreting insulin in response to glucose through the recruitment of GLP-1R and preventing hypoglycemic episodes, GLP-1 analogs that are resistant to proteolytic inactivation by DPP-4 are capable of restoring the β-cell mass and overall function of the pancreas (Parsi et al., 2025), which is an advantage of GLP-1RAs over other conventional therapeutic classes. Therefore, several peptide-based GLP-1 analogs, such as Semaglutide and Tirzepatide (Rosenstock et al., 2021; Karagiannis et al., 2022), which have slower elimination kinetics, are currently marketed as GLP-1RAs for the management of T2DM. Despite the success of peptide-based GLP-1RAs, small-molecule mimetics of GLP-1 have also been developed to target the GLP-1R for insulin exocytosis. For instance, one such non-peptide-based GLP-1RA formulation, called Orforglipron (LY3502970), has been developed and is currently undergoing Phase III clinical trials (Wharton et al., 2025; Horn et al., 2026) as a once-daily oral medication for the treatment of obesity and T2DM. In this context, in reference to GLP-1, the current study comparatively explores the agonist-like properties of a first-generation intermediate-sized designer peptide-based GLP-1RA (SR18), composed of both coded and NCAAs, with the option for future side-chain modification to enhance its *in vivo* circulation time through specific binding to serum albumin. A schematic illustration of the binding characteristics and comparative molecular interaction patterns of GLP-1 and SR18 with GLP-1R is presented in Figure 14.

The available structural data on the GLP-1−GLP-1R system reveal that GLP-1 occupies the deep transmembrane pocket of the GLP-1R and establishes several intermolecular interactions with crucial residues on the N-terminus, ECL2, TM1, TM2, TM5, TM6, and TM7 of GLP-1R (Figure 14), which are also sustained over the duration of MD simulations. For instance, H7 of GLP-1 forms hydrogen and hydrophobic interactions with TM3 and TM5 residues like Y^241^, I^309^, R^310^, and I^313^, whereas A8 forms hydrophobic interactions with L^384^ and L^388^ of TM7. Similarly, H1 of SR18 also establishes interactions with *R* ^299^, W^306^, E^373^, A^375^, and R^376^ of GLP-1R. Notably, alanine substitution studies indicate that *R* ^299^, W^306^, and E^373^ of GLP-1R contribute to the binding affinity and potency of GLP-1. Additionally, mutational studies suggest that L^388^ is an important residue for GLP-1 signaling. Interestingly, Igl2 of SR18 also makes hydrophobic interactions with these two residues. Further, E9 of GLP-1 forms electrostatic and hydrogen bond interactions, respectively, with Y^152^ (TM1) and R^190^ (TM2), which are crucial for GLP-1R activation and signaling. Furthermore, K^197^, a highly conserved residue of GLP-1R, makes hydrogen bond interactions with T13 of GLP-1 (Darbalaei et al., 2023; Li et al., 2023). Notably, E3 and T7 of SR18 also form similar interactions with K^197^ of GLP-1R (Figure 12). Additionally, S14, S17, and S18 of GLP-1 interact with the residues of ECL2 like C^296^, W^297^, T^298^, *R* ^299^and N^300^, whereas no such interactions are observed for SR18. Alanine scanning studies have reported that mutations of residues in the ECL3 and TM6-TM7 region also reduce GLP-1 affinity (Wootten et al., 2016; Lei et al., 2018). Interestingly, T5 and D9 of SR18 establish electrostatic and hydrogen-bonding interactions with R^376^ of ECL3 of GLP-1R. Similarly, H7, A8 and F12 of GLP-1 interact with E^364^ and F^367^ of TM6 of GLP-1R, whereas, Igl2 of SR18 forms hydrophobic interactions with F^367^. In addition, both peptides form multiple hydrophobic interactions with the TM1 residues of GLP-1R. GLP-1 interacts with L^142^, L^145^, and Y^152^, whereas, SR18 interacts with L^141^, L^142^, L^145^, and I^146^ of GLP-1R. Furthermore, the C-terminal part of both peptides makes stable interactions with the N-terminal residues of GLP-1R, which collectively support the potential of SR18 as a GLP-1RA.

**FIGURE 15:**
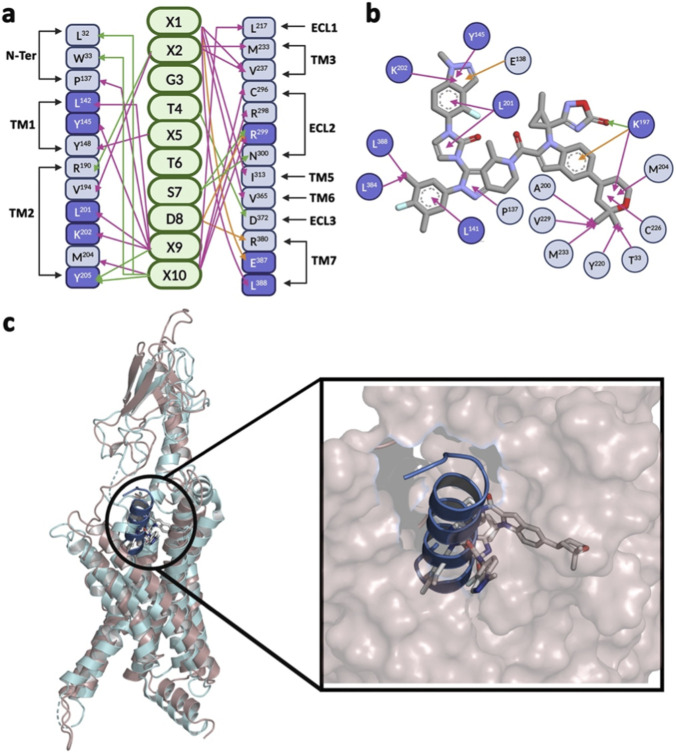
**(a)** Interaction map of peptide 5 with GLP-1R based on PDB: 5NX2. “X” represents a chemically modified amino acid. **(b)** Interaction map of Orforglipron with GLP-1R based on PDB: 6XOX. **(c)** Structural superposition (RMSD: 3.47 Å) of the central conformer of SR18−GLP-1R complex with Orforglipron−GLP-1R complex (cyan) highlights the involvement of similar binding sites. SR18 also recruits the GLP-1R residues highlighted in purple shades. The green line indicates hydrogen bonding, the pink line indicates hydrophobic interactions, and the orange line indicates electrostatic interactions.

Moreover, as illustrated in Figure 15, the binding pattern of SR18 with GLP-1R resembles that of two other reported GLP-1RAs, such as Orforglipron (LY3502970) and peptide 5 (10 aa). Interestingly, GLP-1R residues, such as L^141^, Y^145^, K^197^, L^201^, K^202^, Y^205^, *R* ^299^, L^384^, E^387^, and L^388^, which interacts with these agonists also establish contacts with SR18. Notably, Orforglipron (EC_50_ ∼1.2 nM), a pyrazolopyridine derivative that acts as a biased partial agonist and is administered orally once daily, has entered Phase III clinical trial (NCT3502970). The initial clinical data are encouraging, indicating that Orforglipron produces desirable hypoglycemic activity and also induces weight loss in trial subjects. The three moieties of Orforglipron, 2,2-dimethyl-tetrahydropyran, 3,5-dimethyl-4-fluoro-phenyl ring, and 4H-1,2,4-oxadiazol-5-one, are involved in engaging different segments of the GLP-1R (Kawai et al., 2020), such as the ECD, ECL2, and TMs.

**FIGURE 16:**
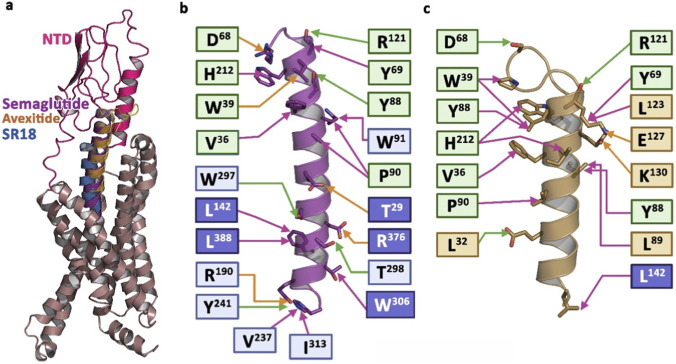
**(a)** Structural superposition of the SR18 (blue), Semaglutide (pink), and Avexitide (brown) highlights the distinct binding interactions with the loop remodeled GLP-1R. **(b)** Interaction map of Semaglutide with loop remodeled GLP-1R. **(c)** Interaction map of Avexitide with loop remodeled GLP-1R. The GLP-1R residues highlighted in green shades are common to both Semaglutide and Avexitide, whereas the residues highlighted in purple shades are common to SR18. The green line indicates hydrogen bonding, the pink line indicates hydrophobic interactions, and the orange line indicates electrostatic interactions.

On the other hand, the further development of Danuglipron (PF-06882,961), another orally active and potent small-molecule GLP-1RA, was discontinued recently due to reported adverse effects in Phase II clinical trials (Buckeridge et al., 2025). Further, Peptide 5, a truncated decapeptide analog of GLP-1 produced after modifying the residues (shown as X) 7–17 of GLP-1, has earlier been shown to have binding affinity and potency similar to full-length GLP-1. Interestingly, Peptide 5, which contains several modified amino acids, also harbors an α-helical conformation (Jazayeri et al., 2017) similar to SR18. Additionally, molecular docking suggests that SR18 binds deep into the active site of loop remodeled GLP-1R, similar to Semaglutide, whereas Avexitide strongly interacts with the NTD of the GLP-1R (Figure 16). Moreover, the binding of both Semaglutide and Avexitide involves several common residues (highlighted in green shades) of GLP-1R. On the contrary, SR18 recruits only one residue of GLP-1R that is involved in binding to Avexitide. Interestingly, SR18 establishes contact with five common residues (highlighted in purple shades) of GLP-1R involved in the binding of Semaglutide (Figure 16). Therefore, it is highly likely that SR18 will be able to produce the desired agonistic effect by binding to the GLP-1R under experimental conditions.

**FIGURE 17:**
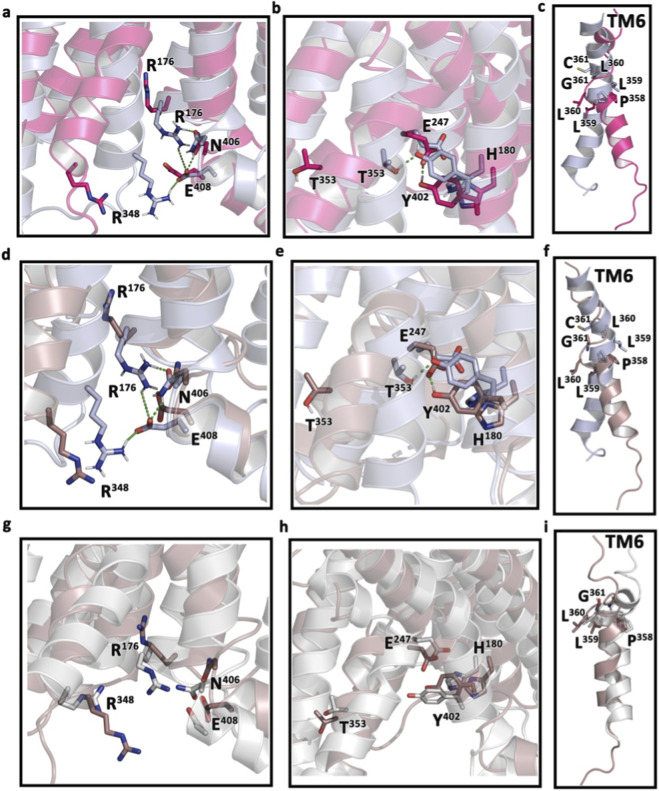
Comparison of the conserved motifs through structural superposition of the active (central conformer, pink) and inactive GLP-1R (sky blue, PDB: 6LN2). **(a)** The disruption of the central hydrogen bond network (TM2-TM6-TM7-H8) observed in the GLP-1−GLP-1R complex compared to the GLP-1 free inactive GLP-1R. **(b)** The reorganization of the HETX motif (TM2–TM3–TM6–TM7) observed in the GLP-1−GLP-1R complex compared to the inactive GLP-1R. **(c)** The rearrangement of the PXXG motif (Gly = Cys in the inactive structure) leading to the movement of TM6, observed in the GLP-1−GLP-1R complex, compared to the inactive GLP-1R. Similar observations made in the SR18−GLP-1R complex are presented in **(d-f)**, respectively. Structural superposition of conserved motifs of semaglutide-GLP-1R complex (gray, PDB: 7KI0) with SR18−GLP-1R complex is further presented in **(g-i)**, respectively.

It is well established that ligand binding may not always trigger the receptor signaling. Therefore, it was essential to extract information from MD simulation data to understand whether the binding of SR18 alters the signaling switches of GLP-1R compared to its reported ligand-free structure. Similar to the E/DRY motif of class A GPCR, Class B GPCRs contain several highly conserved transmembrane polar residues, such as R190^2.60^, N240^3.43^, H363^6.52^, and Q394^7.49^ that form the central polar networks (Figure 17) involving TM2–TM3–TM6–TM7 (H180^2.50^, E247^3.50^, T353^6.42^, Y402^7.57^) and TM2-TM6-TM7-H8 (R176^2.46^, R348^6.37^, N406^7.61^, E408^8.49^) at the cytoplasmic face that lock the base of the receptors in the inactive state (Wootten et al., 2013). Furthermore, a cluster of conserved hydrophobic residues, located between the central hydrogen bond network and the TM2–TM3–TM6–TM7 network, helps in stabilizing the P^6.47^XXG^6.50^ motif (TM6) in the inactive conformation (Cary et al., 2023). It is described that the PXXG motif is susceptible to unwinding due to the presence of proline and glycine residues, which are crucial for receptor activation and signal transduction. Binding of GLP-1 reorganizes the central hydrogen bond network (R176^2.46^, R348^6.37^, N406^7.61^, E408^8.49^), including the destabilization of the P^6.47^XXG^6.50^ motif, which facilitates the movement of TM6, leading to the binding of the G protein and activation of the GLP-1R (Figure 17). In this process, Y^7.57^ and H^2.50^ change their inactive rotameric state and reorganize to form part of the hydrophobic network. As a result, E^3.50^ engages Y^7.57^ with a hydrogen bond, stabilizing the active state of the receptor (Liang et al., 2018; Zhang et al., 2020). Notably, similar to the native GLP-1, binding of SR18 to GLP-1R also induces structural changes in the conserved motifs (Figure 17) in comparison to the inactive (PDB: 6LN2) GLP-1R (Wu et al., 2020), which is crucial for intracellular signaling of GLP-1R. Additionally, structural superposition of the SR18-GLP-1R complex with the Semaglutide complexed to GLP-1R reasonably suggests that SR18 has strong potential to act as an agonist of GLP-1R. However, the agonist like properties of SR18 hypothesized in the current study needs to be evaluated and established further experimentally in appropriate biological systems in the future.

## 5 Conclusion

The expression of GLP-1R in tissues other than the pancreas, such as the brain, endocrine tissues, heart muscle, kidneys, lungs, muscle, and vascular tissues, as well as the proximal digestive and gastrointestinal tract, indicates that GLP-1 has a multifaceted regulatory role beyond glucose homeostasis mediated by insulin release. The broad expression profile of GLP-1R benefits the reported GLP-1RAs to combat various T2DM-associated comorbidities. Notably, GLP-1 signaling helps regulate higher-order central nervous system functions, including learning and memory, reward processing, and food palatability. Additionally, native GLP-1 increases endothelial function and vasodilation in both diabetic and non-diabetic patients, regardless of alterations in insulin sensitivity. Furthermore, GLP-1 exhibits significant cardioprotective properties, particularly in pathological conditions, such as ischemia and myocardial damage. Notably, GLP-1 analogs that can pass the BBB have been shown to produce strong neuroprotective effects in preclinical models of Alzheimer’s disease, Parkinson’s disease, stroke, and other neurological conditions. Interestingly, GLP-1 signaling in the hippocampus has been described to play a direct and crucial role in regulating learning and memory, independent of motor, stress, or nociceptive impacts. Overall, it is evident that GLP-1 plays an integrative role in regulating feeding behavior, synchronizing neuronal circuits linked to metabolism, stress, and reward. Therefore, the design, discovery, and development of short and long-acting peptide-based GLP-1RAs, including small-molecule agonists are desirable for therapeutic applications beyond T2DM.

On the other hand, certain studies have demonstrated the overexpression of GLP-1R in brain tumors, embryonic tumors, gut and lung neuroendocrine tumors, including other endocrine tumors like Pheochromocytomas, raising concerns that existing GLP-1RAs can contribute to tumor growth in humans (Korner et al., 2007; Shilyansky et al., 2025; Ungvari et al., 2025). In this context, the functional mimicry of GLP-1 should be considered worthwhile if the designed agonist can act as a biased agonist of GLP-1R, selectively triggering anti-inflammatory signaling pathways that can aid in reducing the growth and differentiation of certain types of neuroendocrine tumors. Furthermore, excessive insulin secretion in the case of congenital as well as acquired hyperinsulinism patients can also cause clinically fatal outcomes if it remains uncontrolled (Stefanovski et al., 2022; Peterson et al., 2023). Therefore, designer peptides that can act as GLP-1R antagonists to modulate excessive insulin in the bloodstream also have therapeutic value. The rational design of a potential medium-sized GLP-1RA, such as SR18, described in the current study, opens up the aforementioned avenues for future explorations.

## Data Availability

The original contributions presented in the study are included in the article/Supplementary Material, further inquiries can be directed to the corresponding author.
