# Supplementary material for: A rationally designed 18-amino acid peptide with potential as GLP-1 receptor agonist
Source: Front Pharmacol. 2026 May 20;17:1789257. doi: 10.3389/fphar.2026.1789257 (PMC13229714; doi:10.3389/fphar.2026.1789257)
Supplement: Supplementary file 1 [file Supplementaryfile1.pdf]

Supplementary Information

A Rationally Designed 18-amino acid Peptide with Strong Potential as GLP-1 Receptor Agonist

Aditi Singh[1], Sucharita Shadangi[1], and Soumendra Rana*[1]

[1]Chemical Biology Laboratory, School of Basic Sciences, Indian Institute of Technology Bhubaneswar, Odisha 752050, India. Email: soumendra@iitbbs.ac.in

Contents		Page
**Figure S1**	Monitoring the sustainability of intermolecular (hydrogen, electrostatic, and hydrophobic) interactions observed between GLP-1 and GLP-1R throughout the MD simulation. The GLP-1R residues are shown in superscript. The grey line highlights the cutoff distance for the specified interaction between the amino acids.	**3**
**Figure S2**	Structural representation of the lipid moiety of Semaglutide at K26 and SR18 at K17.	**3**
**Figure S3**	Comparative graph showing (a) major conformational clusters populated for GLP-1 and SR18 in MD simulation. Intramolecular hydrogen bond for (b) GLP-1 and (c) SR18. GLP-1 maintains an average of 18 intramolecular hydrogen bonds, whereas SR18 maintains an average of 12 intramolecular hydrogen bonds.	**4**
**Figure S4**	The ESI-MS spectra and analytical HPLC profile (inset) of SR18.	**4**
**Figure S5**	DLS analysis of 10 µM BSA in 1X PBS.	**4**
**Figure S6**	HRMS spectra of SR4 in the (a) absence and (b) presence of trypsin. The respective chromatograms are provided as insets. The digested fragments are highlighted with asterisks.	**5**
**Figure S7**	HRMS spectra of SR18 in the (a) absence and (b) presence of trypsin. The respective chromatograms are provided as insets. The digested fragments are highlighted with asterisks.	**5**
**Figure S8**	Monitoring the sustainability of intermolecular (hydrogen, electrostatic, and hydrophobic) interactions observed between SR18 and GLP-1R throughout the MD simulation. The GLP-1R residues are shown in superscript. The grey line highlights the cutoff distance for the specified interaction between the amino acids.	**6**

**Table S1**	Docking Score of the Designer Helical Peptides	**6-7**
**Table S2**	α-Helix Content Calculation from CD Data	**7**

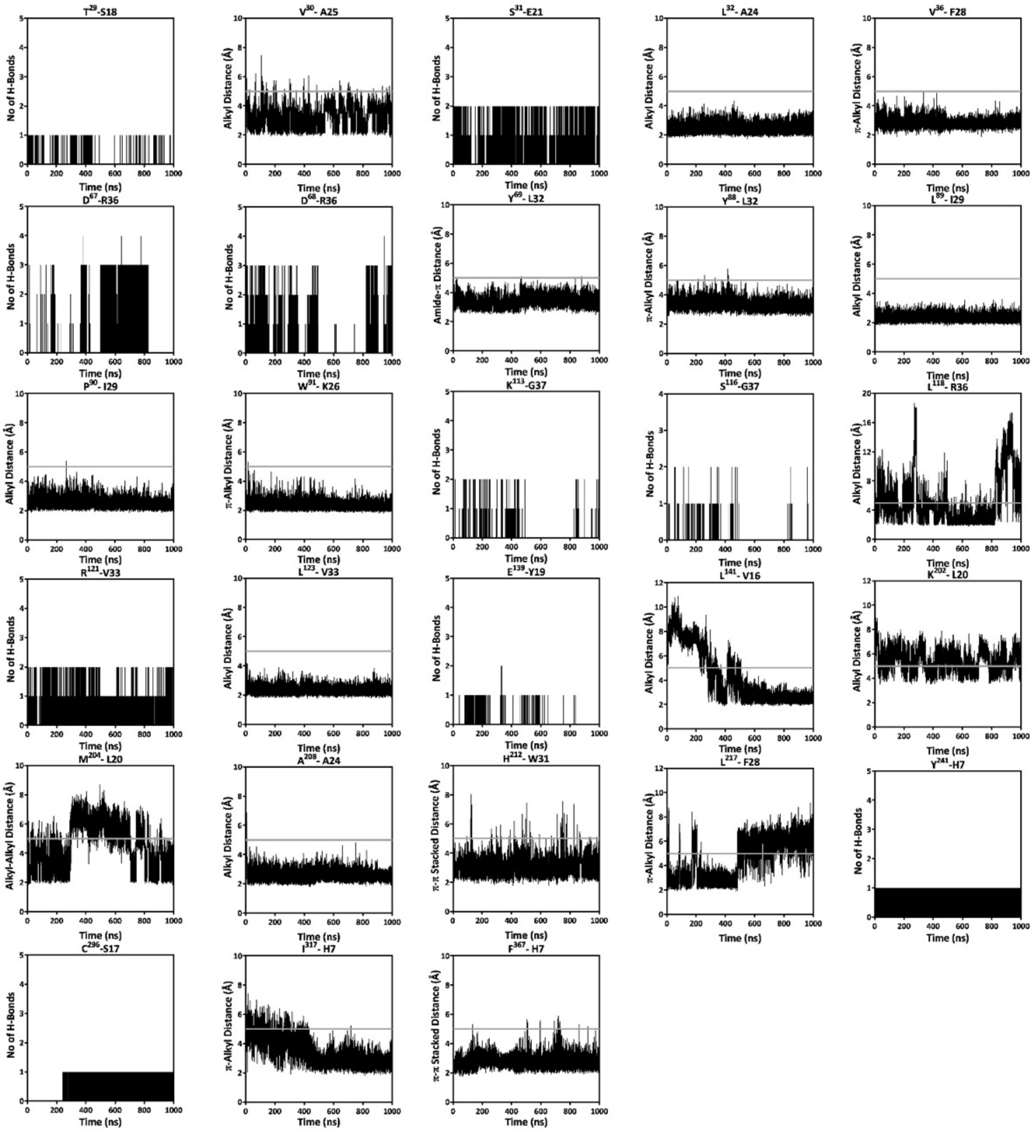

Figure S1: Monitoring the sustainability of intermolecular (hydrogen, electrostatic, and hydrophobic) interactions observed between GLP-1 and GLP-1R throughout the MD simulation. The GLP-1R residues are shown in superscript. The grey line highlights the cutoff distance for the specified interaction between the amino acids.

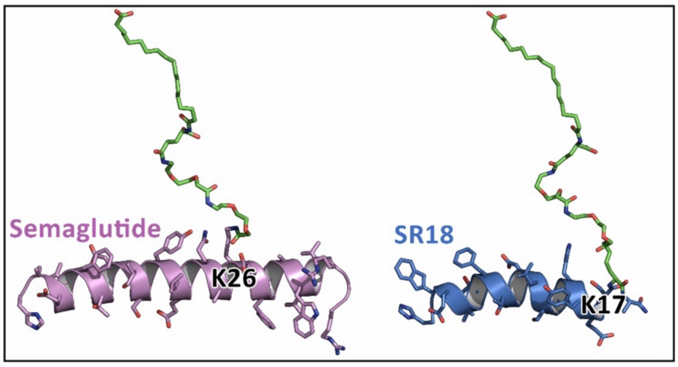

Figure S2: Structural representation of the lipid moiety of Semaglutide at K26 and SR18 at K17.

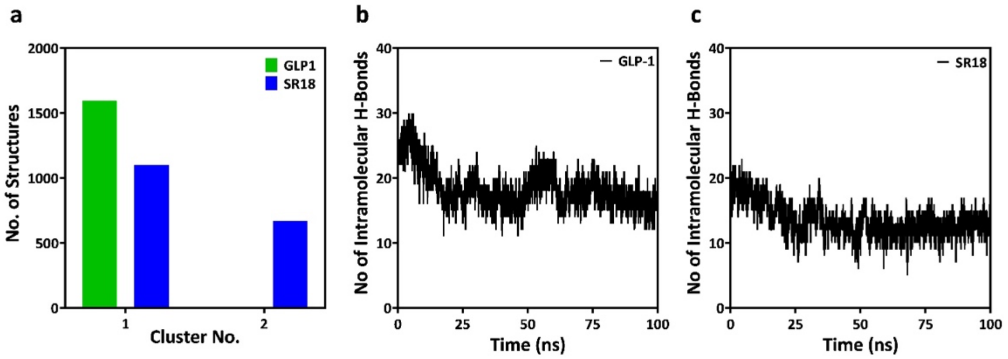

Figure S3: Comparative graph showing intramolecular hydrogen bond for (a) GLP-1 and (b) SR18. GLP-1 maintains an average of 18 intramolecular hydrogen bonds, whereas SR18 maintains an average of 12 intramolecular hydrogen bonds.

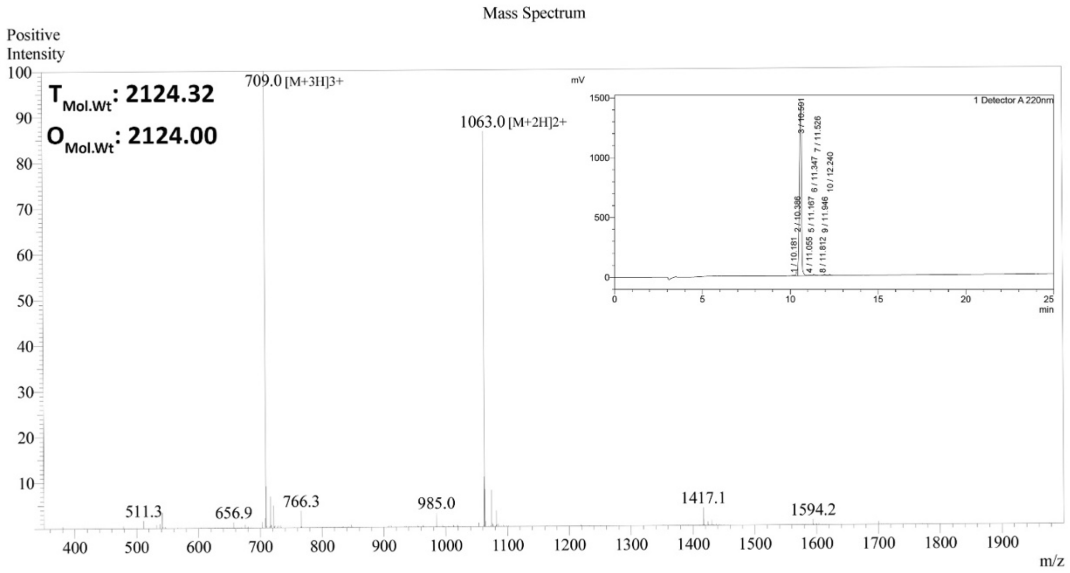

Figure S4: The ESI-MS spectra and analytical HPLC profile (inset) of SR18.

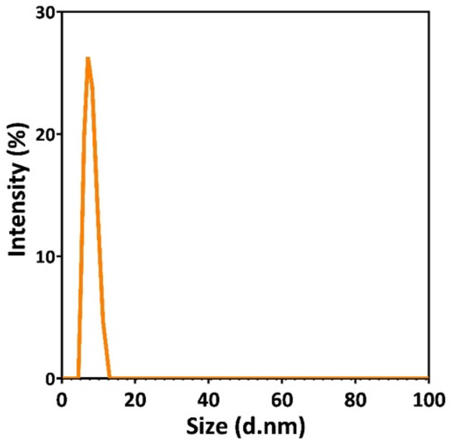

Figure S5: DLS analysis of 10 μM BSA in 1X PBS.

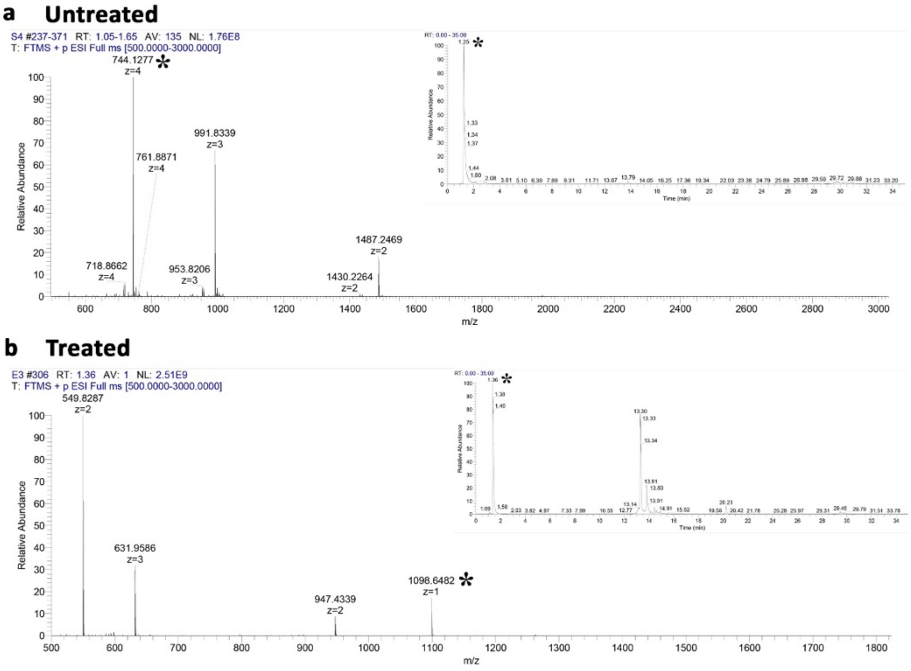

Figure S6: HRMS spectra of SR4 in the (a) absence and (b) presence of trypsin. The respective chromatograms are provided as insets. The digested fragments are highlighted with asterisks.

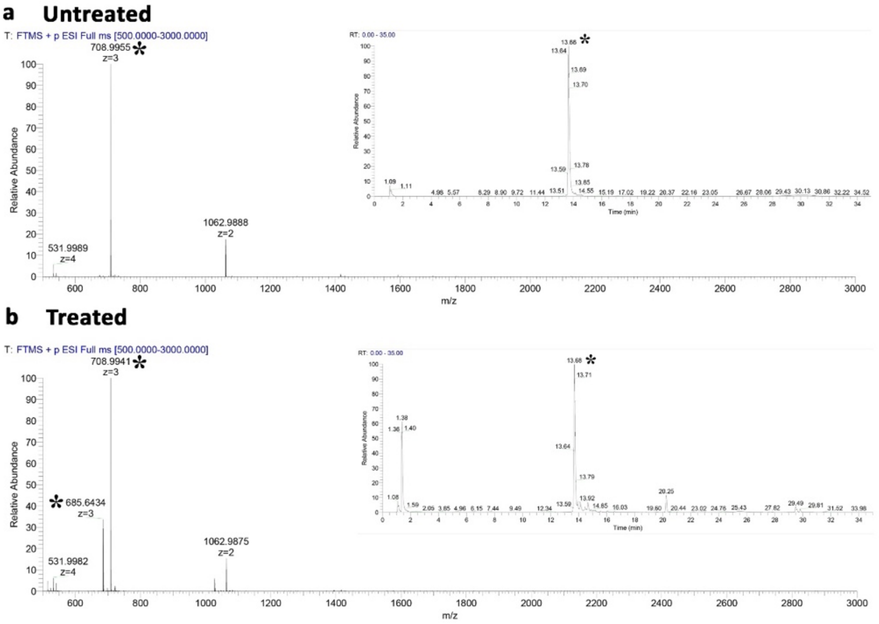

Figure S7: HRMS spectra of SR18 in the (a) absence and (b) presence of trypsin. The respective chromatograms are provided as insets. The digested fragments are highlighted with asterisks.

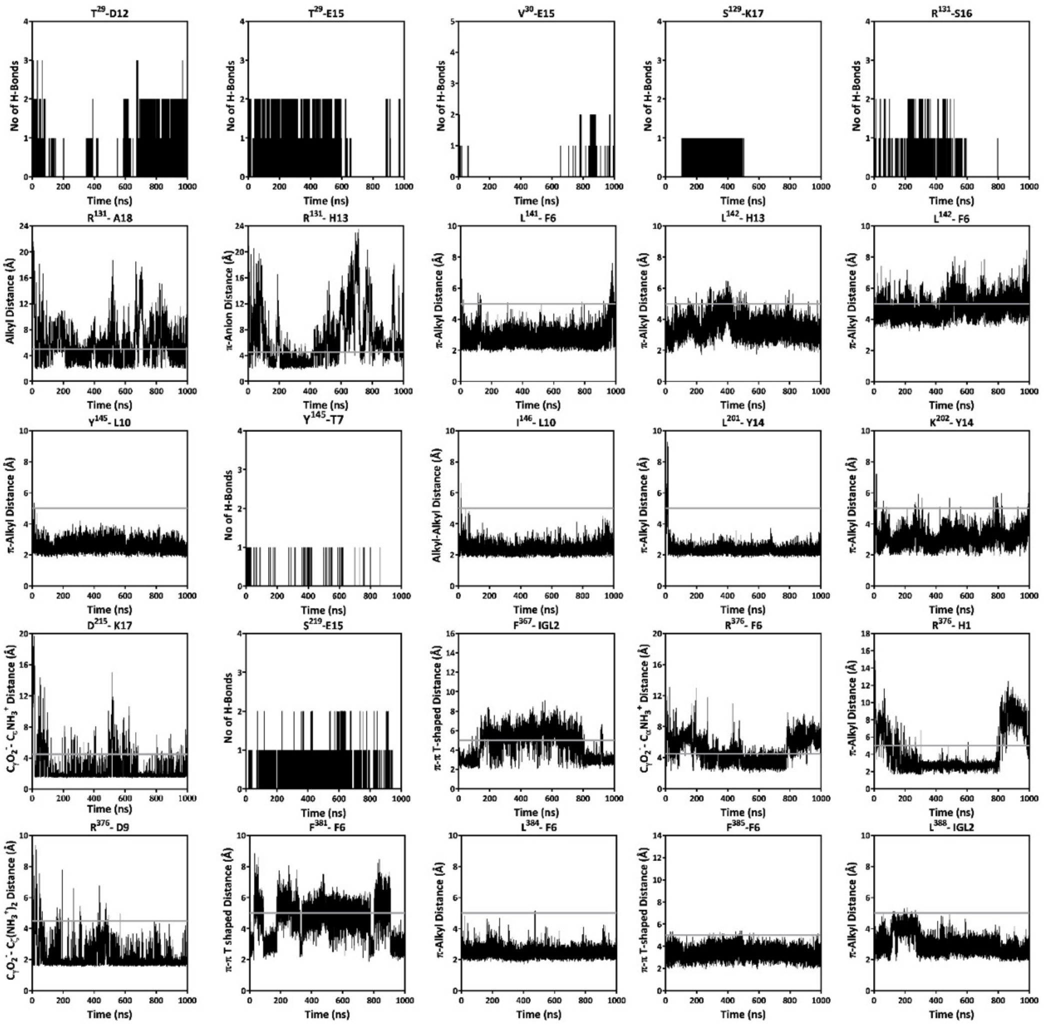

Figure S8: Monitoring the sustainability of intermolecular (hydrogen, electrostatic, and hydrophobic) interactions observed between SR18 and GLP-1R throughout the MD simulation. The GLP-1R residues are shown in superscript. The grey line highlights the cutoff distance for the specified interaction between the amino acids.

Table S1: Docking Score of the Designer Helical Peptides

S.NO	Peptides	Score	Confidence Score	Ligand RMSD
**1.**	Hed95	-352.98	0.9830	10.06
**2.**	Hed178	-335.80	0.9762	10.53
**3.**	Hed102	-319.17	0.9672	10.55
**4.**	Hed 220	-314.95	0.9644	17.99
**5.**	Hed87	-312.55	0.9627	10.87
**6.**	Hed94	-303.88	0.9560	8.72
**7.**	Hed167	-294.65	0.9475	26.32
**8.**	Hed13	-294.16	0.9470	14.29
**9.**	Hed153	-292.97	0.9458	25.08
**10.**	Hed156	-291.50	0.9443	9.76
**11.**	Hed7	-290.50	0.9432	21.04
**12.**	Hed176	-289.54	0.9422	9.88

**13.**	Hed155	-288.05	0.9405	17.35
**14.**	Hed51	-285.72	0.9379	13.97
**15.**	Hed119	-285.57	0.9377	9.22
**16.**	Hed22	-280.79	0.9319	10.11
**17.**	Hed37	-280.49	0.9315	13.40
**18.**	Hed132	-279.64	0.9304	13.89
**19.**	Hed103	-273.68	0.9223	11.89
**20.**	Hed195	-272.52	0.9206	10.63
**21.**	Hed201	-272.47	0.9205	16.95
**22.**	**Hed196 (SR18)**	**-272.14**	**0.9209**	**8.09**
**23.**	Hed162	-269.04	0.9154	17.26
**24.**	Hed172	-268.12	0.9139	10.56
**25.**	Hed9	-267.44	0.9128	15.36
**26.**	Hed65	-266.10	0.9107	9.50
**27.**	Hed23	-264.62	0.9082	17.82
**28.**	Hed211	-257.94	0.8965	17.89
**29.**	Hed15	-256.36	0.8935	17.65
**30.**	Hed209	-254.27	0.8895	9.70
**31.**	Hed25	-254.01	0.8890	24.91
**32.**	Hed177	-253.55	0.8881	15.67
**33.**	Hed29	-251.51	0.8839	17.26
**34.**	Hed71	-243.96	0.8675	20.26
**35.**	Hed219	-242.77	0.8648	18.31
**36.**	Hed187	-236.19	0.8486	17.90
**37.**	Hed137	-235.25	0.8462	13.67
**38.**	Hed143	-230.97	0.8347	12.18
**39.**	Hed215	-226.77	0.8228	13.95
**40.**	Hed 141	-208.58	0.7634	17.92
**41.**	Hed61	-202.07	0.7391	10.55

Table S2: α-Helix Content Calculation from CD Data

**Solvents**	**CDNN Prediction (190-260 nm)**	$[\theta_{Exp}]_{222}/[\theta_{Theor}]_{222}$
20 % TFE	41.0 %	0.12
30 mM SDS	35.3 %	0.17
20 % HFIP	34.1 %	0.22
1 X PBS	28.6 %	0.034
